# Non-catalytic signaling by pseudokinase ILK for regulating cell adhesion

**DOI:** 10.1038/s41467-018-06906-7

**Published:** 2018-10-26

**Authors:** Julia Vaynberg, Koichi Fukuda, Fan Lu, Katarzyna Bialkowska, Yinghua Chen, Edward F. Plow, Jun Qin

**Affiliations:** 1 Department of Molecular Cardiology, Lerner Research Institute, Cleveland Clinic, 9500 Euclid Avenue, Cleveland, OH 44195 USA; 2 Department of Biochemistry, School of Medicine, Case Western Reserve University, Cleveland, OH 44106 USA; 3 Department of Physiology and Biophysics, School of Medicine, Case Western Reserve University, Cleveland, OH 44106 USA

## Abstract

Dynamic communication between integrin-containing complexes (focal adhesions, FAs) and actin filaments is critical for regulating cell adhesion. Pseudokinase ILK plays a key role in this process but the underlying mechanism remains highly elusive. Here we show that by recruiting FA adaptors PINCH and Parvin into a heterotrimeric complex (IPP), ILK triggers F-actin filament bundling – a process known to generate force/mechanical signal to promote cytoskeleton reassembly and dynamic cell adhesion. Structural, biochemical, and functional analyses revealed that the F-actin bundling is orchestrated by two previously unrecognized WASP-Homology-2 actin binding motifs within IPP, one from PINCH and the other from Parvin. Strikingly, this process is also sensitized to Mg-ATP bound to the pseudoactive site of ILK and its dysregulation severely impairs stress fibers formation, cell spreading, and migration. These data identify a crucial mechanism for ILK, highlighting its uniqueness as a pseudokinase to transduce non-catalytic signal and regulate cell adhesion.

## Abstract (web summary)

Pseudokinase ILK is essential for dynamic communication between focal adhesion complexes and F-actin filaments. Here, the authors show that ILK regulates such communication by complexing with PINCH and Parvin to trigger F-actin filament bundling through previously unrecognized actin binding motifs in PINCH and Parvin

## Introduction

**Fig. 1:**
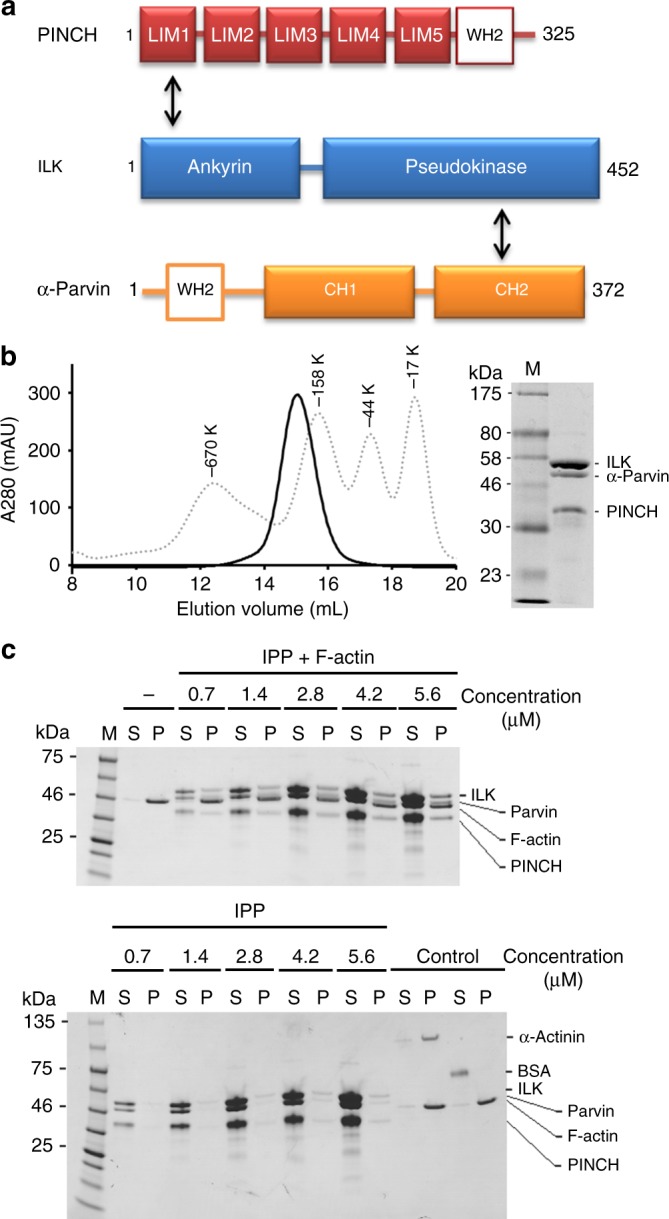
IPP interaction with F-actin. **a** Schematic organization of IPP based on structural data. ILK binds to PINCH LIM1 via its ankyrin domain and α-Parvin CH2 via its pseudokinase domain, respectively. The Wiscott–Aldrich syndrome protein (WASP) homology domain (WH2) motifs are highlighted in PINCH and α-Parvin. **b** A representative gel filtration profile of the purified IPP complex by Superose 6 10/300 GL size exclusion chromatography column (GE healthcare). The eluted peak is overlaid with an elution curve of standard molecular weight proteins (dot lines). **c** Co-sedimentation of IPP at dose-dependent amounts in the presence/absence of F-actin. The F-actin was incubated at 2.3 μM constant concentration with increasing concentrations of each test sample in 5% glycerol containing protein buffer. Representative gels with Coomassie stain are shown. M marker proteins, S supernatant, P pellets

The adhesion of cells to extracellular matrix (ECM) is a fundamental step for controlling diverse physiological processes such as blood clotting, hemostasis, host defense, and tissue regeneration. The adhesion is mediated by heterodimeric (α/β) integrin transmembrane receptors that bind to ECM proteins. However, for cells to firmly attach, ECM must physically connect to intracellular actin cytoskeleton via integrin-containing protein complexes called focal adhesions (FAs)^1–4^. Integrin-linked kinase (ILK) is one of the few evolutionarily conserved proteins found in FAs to critically control the FA assembly and integrin–actin connection^5^. Discovered two decades ago^6^, ILK was originally thought to act as a Ser/Thr kinase to phosphorylate integrin β cytoplasmic tail and other targets to promote the integrin–actin communication, regulating dynamic cell adhesion events such as cell spreading and migration^7^. However, sequence analysis suggested that despite containing kinase-like domain, ILK is a pseudokinase lacking several key active site residues^8^. This triggered extensive genetic^9–12^ and structural^13,14^ studies, which confirmed that ILK is indeed a pseudokinase with distinct scaffolding ability to bind many proteins for regulating cell adhesion and migration^15^. Notably, ILK was found to form a tight obligate ternary complex with FA adaptors PINCH and Parvin (termed IPP thereafter), which occurs early before the localization to FAs^16^. PINCH has two isoforms PINCH-1 and PINCH2, which both contain five LIM domains whereas Parvin has three isoforms, α-, β-, γ-Parvin, which all contain two calponin homology (CH) domains^5,7,15^. These isoforms form cell-type specific IPPs to regulate dynamic integrin–actin connection, dysfunctions of which were linked to many diseases including cancer, diabetes, and heart failure^5,7,15,17,18^. Detailed structural analyses revealed that the N-terminal ankyrin repeat domain (ARD) of ILK recognizes PINCH LIM1^19–22^, whereas C-terminal kinase-like domain (KLD) of ILK specifically binds Parvin CH2 (Fig. 1a)^13,14,22^, thereby allowing the tight IPP complex formation^13^.

While ILK is now widely recognized as the pseudokinase^15,18,23^, a fundamental issue still remains unresolved: without catalytic function, how could ILK mediate the integrin–actin communication to promote diverse cell adhesive processes? ILK is clearly indispensable for this dynamic signaling event as evidenced by mounting genetic and cell biological data^5,7,15,17,23^. In this study, we have undertaken a combination of structural, biochemical, and cell biological studies to address this issue. Our results reveal that by recruiting FA adaptors PINCH and Parvin into a heterotrimeric complex (IPP), ILK is able to trigger F-actin filament bundling via two WASP-Homology-2 actin-binding motifs, one from PINCH and the other from Parvin. We further show that this process is sensitized to Mg-ATP bound to the pseudoactive site of ILK and its dysregulation severely impairs stress fibers formation, cell spreading, and migration. Our data identify a crucial mechanism for ILK, highlighting its uniqueness as a pseudokinase to transduce non-catalytic signal and regulate cell adhesion.

## Results

### IPP binds to F-actin via an unexpected manner

To address the above issues, we decided to focus on IPP—the major functional form of ILK crucial for the integrin–actin connection^5,7,15,17,18^. To begin with, we decided to examine a hypothesis that IPP physically connects integrin and actin filaments to promote mechanical regulation of integrin–actin linkage. This hypothesis was derived from the data suggesting that ILK KLD contains the integrin binding site^6^ and Parvin contains putative F-actin-binding CHs^5,15,18^. However, while ILK KLD indeed binds integrin β cytoplasmic tails^6,13^, purified CH1–CH2 of α-Parvin had essentially no interaction with F-actin in our co-sedimentation assay (Supplementary Figure 1A). CH domains in Parvin have sequences that diverge significantly from typical F-actin binding CH domain^24^, suggesting that they may have evolved to become nonactin-binding modules, e.g., Parvin CH2 was shown to bind paxillin and ILK^13,25^. Given the crucial role of IPP in actin cytoskeleton, we then wondered if IPP would engage F-actin via other regions of the complex. To test this possibility, we coexpressed and purified recombinant full-length ILK, PINCH-1, and α-Parvin with the latter two being the major PINCH and Parvin isoforms, respectively^5^. Size exclusion chromatography revealed that the three proteins co-eluted at an expected molecular mass (~133 kDa) corresponding to the tight ternary IPP complex with relatively elongated stokes radius (Fig. 1b). Interestingly, intact IPP complex potently associated with F-actin (Fig. 1c and Supplementary Figure 1B). These data thus indicate that IPP binds to F-actin yet in a previously unrecognized manner.

### IPP binds F-actin via two WH2 motifs in PINCH and Parvin

**Fig. 2:**
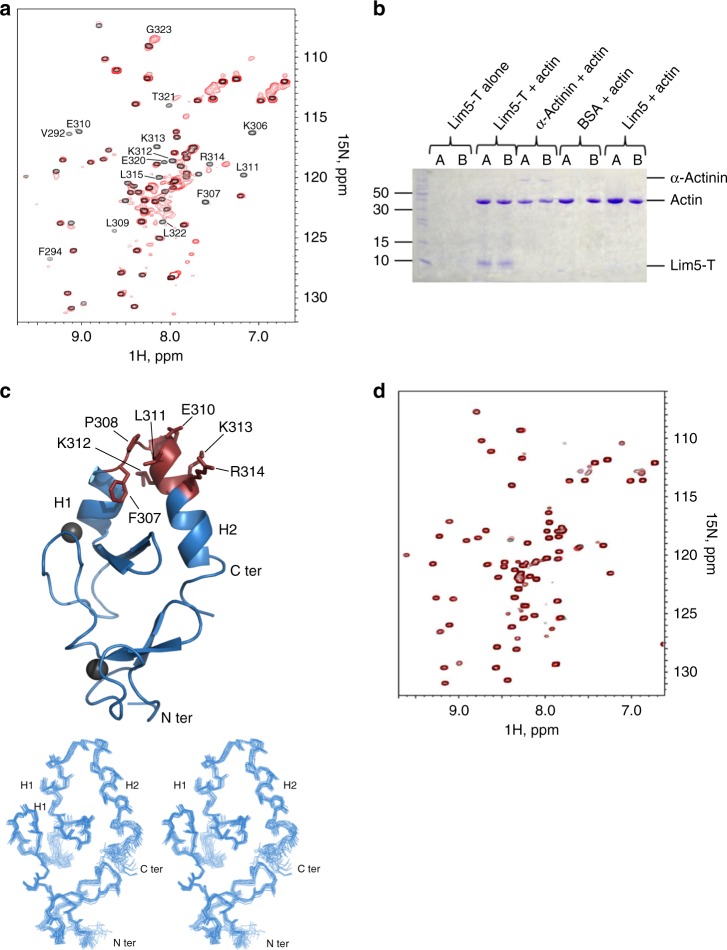
Mapping and structural analysis of the major actin binding site in PINCH-1. **a** Overall, 0.1 mM ^1^H-^15^N HSQC of LIM5-T in the absence (black) and presence (red) of 0.2 mM AP-actin showing that LIM5-T binds to G-actin. Strongly perturbed residues in LIM5-T by the actin binding are labeled, which primarily involve PINCH-1 C-terminal tail. **b** Co-sedimentation data (pellets in duplicates) showing LIM5-T but not LIM5 alone binds to F-actin. **c** (Top) NMR structure of LIM5-T and the actin binding surface derived from the chemical shift mapping. Note that the H2 helix that packs against the LIM domain as reflected in all calculated structures. Residues undergoing the largest chemical shift changes upon interaction with AP-actin are shown with side chains. Two zinc atoms are shown as gray spheres. (Bottom) Superposition of 20 lowest energy structures of LIM5-T. **d** 0.1 mM ^1^H-^15^N HSQC of C-terminal LIM5-T 4A mutant (F307A/L311A/K312A/K313A) in the absence (black) and presence (red) of 0.2 mM AP-actin showing that the mutations drastically reduced the actin binding to LIM5-T

To understand the molecular basis of the IPP-actin binding, we set out to perform systematic biochemical and structural analyses. We first carried out the standard co-sedimentation experiments on each IPP subunit to examine their individual F-actin-binding capacity. Full-length ILK exhibits no binding to F-actin (Supplementary Figure 1C), but interestingly, both full-length PINCH-1 (Supplementary Figure 1D) and α-Parvin bind to F-actin (Supplementary Figure 1A). This result suggested that IPP engages F-actin via a dual binding mode involving PINCH and Parvin, respectively. To further examine this potential binding mode, we employed definitive NMR-based ^1^H-^15^N heteronuclear single quantum coherence spectroscopy (HSQC) to map the actin-binding domains within PINCH or Parvin. We first used nonpolymerizable G-actin mutant (A204E/P243K, termed AP-actin hereafter)^26^ to examine the actin-binding properties of PINCH or Parvin. The nonpolymerizable AP-actin has the size of ~40 kDa suitable for NMR-based binding experiments whereas wild-type G-actin can readily polymerize into large F-actin polymer at concentrations (> 10 μM) typically used in NMR experiments. We divided PINCH-1 into three domain fragments: LIM1-2, LIM3-4, and LIM5-T containing the C-terminal tail. ^15^N-labeled PINCH-1 LIM1-2 and LIM3-4 showed no spectral change upon addition of unlabeled AP-actin (Supplementary Figure 2A and 2B), but ^15^N-labeled PINCH LIM5-T exhibited substantial spectral perturbation upon addition of unlabeled AP-actin, demonstrating that the G-actin-binding site is localized in LIM5-T (Fig. 2a). Next, we examined if LIM5-T also binds F-actin. As expected, the co-sedimentation assay showed that LIM5-T also bound to F-actin (Fig. 2b). By contrast, LIM5 alone without the C-terminal tail did not interact with F-actin (Fig. 2b), indicating that the major actin-binding site is localized in the PINCH-1 C-terminal tail. To further examine the molecular details of the LIM5-T/actin interaction, we determined the solution NMR structure of LIM5-T (Table 1). Figure 2c reveals a canonical LIM fold for LIM5 that contains two zinc finger motifs followed by a short C-terminal helix (H1, K301-K306), which is very similar to other previously reported PINCH-1 LIM1 (PDB code 3F6Q, RMSD 3.5 Å between 49 equivalent Cα atoms of LIM5 residues V251-E305 and Lim1 A192-Q246) and LIM4 (PDB code 1U5S, RMSD 1.5 Å between 52 equivalent Cα atoms of LIM5 residues V250-E304 and LIM4 I192-Q246) structures (Supplementary Figure 2C). Interestingly, the C-terminal tail beyond LIM5 also contains a helix (H2, L309-T321) that packs against both zinc fingers of LIM5, leading to a variant of the LIM fold (Fig. 2c and Supplementary Figure 2C). The residues that underwent significant spectral changes are marked in Fig. 2a, which are all clustered on a surface encompassed by H2 and the loop (F307–P308) connecting to H1 in LIM5. Mutation of four strongly perturbed residues F307A, L311A, K312A, and K313A (termed PINCH-4A) in the PINCH-1 C-terminal tail (Fig. 2c) substantially reduced the LIM5-T binding to AP-actin (Fig. 2d) and F-actin (Fig S2D vs. Supplementary Figure 2E), further demonstrating that the C-terminal tail is the major actin-binding site.

**Table 1:** NMR and refinement statistics for Lim5-T

	Protein
NMR distance and dihedral constraints	2375
Distance constraints	2263
Total NOE	2263
Intraresidue	422
Interresidue	
Sequential (|*i* – *j*| = 1)	627
Medium-range (|*i* – *j*| < 4)	490
Long-range (|*i* – *j*| > 5)	724
Intermolecular	n/a
Hydrogen bonds	n/a
Total dihedral angle restraints	112
ϕ	56
ψ	56
*Structure statistics*	
Violations (mean and s.d.)	
Distance constraints (Å)	0.01865 ±0.00162
Dihedral angle constraints (°)	0.45826 ±0.05425
Max. dihedral angle violation (°)	3
Max. distance constraint violation (Å)	0.3
Deviations from idealized geometry	
Bond lengths (Å)	0.00478 ± 0.00003
Bond angles (°)	0.84656 ± 0.02087
Impropers (°)	1.99750 ± 0.00512
Average pairwise r.m.s. deviation** (Å)	
Heavy	1.013 ± 0.128
Backbone	0.590 ± 0.080

**Pairwise r.m.s. deviation was calculated among 20 refined structures

**Fig. 3:**
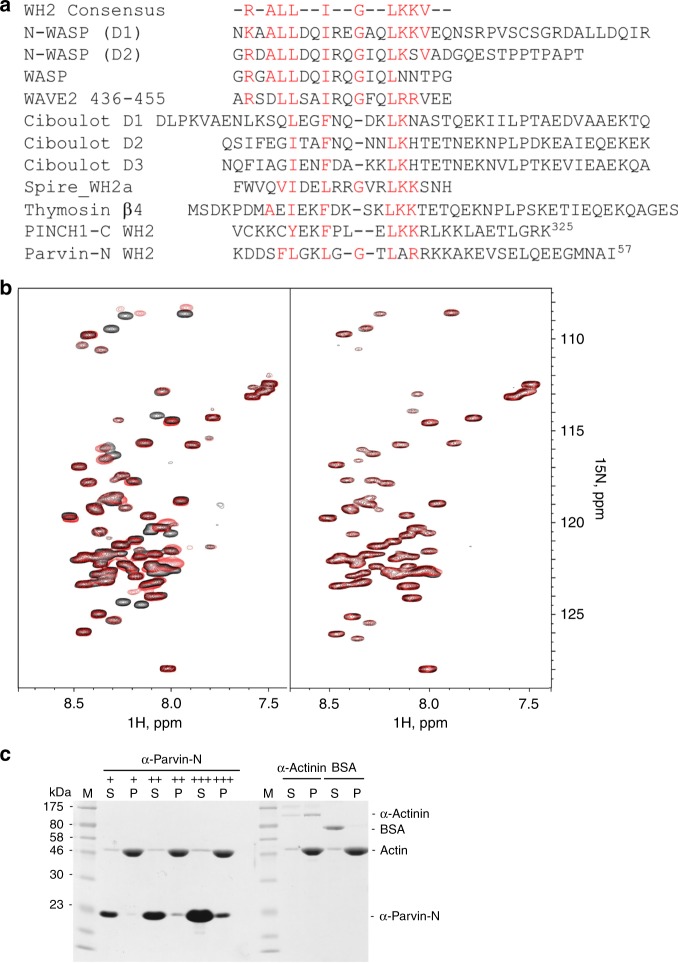
Identification of actin-binding WH2 motifs in PINCH-1 and α-Parvin. **a** Sequence alignment of PINCH-1 C-terminal tail and α--terminus with representative WH2 motifs found in other proteins showing the presence of distinct WH2 motifs in PINCH and Parvin. **b** 0.1 mM ^1^H-^15^N HSQC of α-Parvin-N in the absence and presence of 0.2 mM AP-actin showing that α-Parvin-N has potent binding to G-actin (left panel). Mutation of putative G-actin-binding residues L37A/R39A/R40A/K41A/K42A (α-Parvin 5 A) drastically reduces actin binding (right panel). **c** Co-sedimentation assay showing α-Parvin-N binds to F-actin potently. +, ++, +++ correspond to the concentration of α-Parvin-N at 7.7 μM, 23.0 μM, and 76.7 μM, respectively

Having determined the actin-binding capability of PINCH-1 C-terminal tail, we next wondered if it belongs to any known actin-binding motifs. Interestingly, sequence comparison revealed that the PINCH tail falls into a loosely conserved WASP-Homology 2 (WH2) motif that is known to sequester G-actin^27,28^. Depending on the copy number and specific biochemical conditions, WH2 was also known to regulate F-actin assembly, e.g., promoting actin nucleation and elongation^27,28^ and bundling^29,30^, probably by binding to the G-actin unit of F-actin filament. WH2 motif was suggested to have a consensus sequence of R-ALL--I--G-----LKKV based on 50% of 100 representative WH2 domains^27^. The PINCH-1 C-terminus contains a sequence Y304 (hydrophobic)--F (hydrophobic)---LKK similarly seen in the proposed WH2 consensus sequence (Fig. 3a). Consistently, mutations of F307---LKK in this WH2-like motif to A---AAA significantly reduced the G-actin binding (Fig. 2d) and F-actin binding (Supplementary Figure 2E), suggesting that the PINCH-1 WH2-like motif binds to actin in a manner similar to known WH2s^27^. To further investigate this possibility, we performed HSQC experiments of ^15^N-labeled known WH2 protein thymosin-β4 in the absence and presence of G-actin, which revealed a well-folded thymosin-β4 when bound to G-actin (Supplementary Figure 3A). However, addition of unlabeled LIM5-T into the thymosin-β4/G-actin mixture led to a spectrum that is nearly identical to the free form thymosin-β4 (Supplementary Figure 3A), indicating that thymosin-β4 and LIM5-T compete for binding to a shared G-actin-binding site due to the presence of WH2 motif in both proteins. We note that many residues of H1-loop-H2 of LIM5-T WH2 underwent dramatic line-broadening (K306, F307, and L309-K313) or chemical shift changes (R314–G323) upon addition of G-actin, indicating that this structural motif is significantly involved in binding to G-actin (Fig. 2a) in a manner similar to other WH2/G-actin complexes, e.g., the recent high-resolution structure of Bud6/G-actin complex, where Bud6 WH2 also has helix-loop-helix to interact with G-actin^31^.

To map the actin-binding site on Parvin, we also performed the HSQC experiments on ^15^N-labeled α-Parvin fragments in the absence and presence of AP-actin. Consistent with our earlier co-sedimentation analysis (Supplementary Figure 1A) where α-Parvin CH1–CH2 exhibited little binding to F-actin, α-Parvin CH1–CH2 had little interaction with G-actin (Supplementary Figure 3B). Since full-length α-Parvin bound to F-actin (Supplementary Figure 1A and also see ref. ^32^), we wondered if N-terminal region 1–91 (α-Parvin-N) outside the Parvin CH1–CH2 might be responsible for the actin binding. Figure 3b shows that ^15^N-labeled α-Parvin-N underwent substantial spectral change on a selective set of residues upon addition of AP-actin (Fig. 3b), indicating that α-Parvin-N does contain an actin-binding site. Co-sedimentation experiments showed that α-Parvin-N also associated with F-actin (Fig. 3c). Remarkably, sequence analysis suggested that α-Parvin-N also contains a WH2 motif like PINCH-1 LIM5-T (Fig. 3a). Mutation of the conserved residues L37, R39, R40, K41, and K42 into Ala (named α-Parvin 5 A hereafter) that contains the predicted actin-binding site^27,28^ diminished the α-Parvin-N binding to AP-actin (Fig. 3b), further confirming the specific α-Parvin-N WH2/G-actin interaction. Co-sedimentation assay showed that WT α-Parvin also binds F-actin at the K_D_~21 μM (Supplementary Figure 3C), but as a comparison, the α-Parvin-N 5 A has significantly reduced affinity to F-actin (K_D_~49 μM) (Supplementary Figure 3C). As expected, α-Parvin-N competes with PINCH LIM5-T for binding to AP-actin (Supplementary Figure 3E). Note that α-Parvin-N is largely unstructured based on its narrow chemical shift range (Fig. 3b) like thymosin β4 (Supplementary Figure 3A) so its total structure determination was not pursued but the WH2 motif likely adopts helical conformation when bound to actin as shown by previous studies^27,28^. Indeed, the secondary structure prediction revealed that the α-Parvin-N WH2 (Fig. 3a) contains a helix-loop-helix structural motif (Supplementary Figure 3F), which is similar to that of PINCH WH2 (Figs. 2c and  3a). We note again that the WH2 sequences are loosely conserved, which exhibit a large range of actin-binding affinities, e.g., K_D_ is ~52 nM for WAVE2 WH2 but ~53 μM for Ciboulot D2^32^. WAVE2 WH2 is better aligned with the consensus sequence than Ciboulot D2 (Fig. 3a), which appears to correlate with their actin affinity strengths. Consistently, PINCH/Parvin WH2s are less well aligned with the consensus sequence and thus their actin-binding affinities are closer to those of Ciboulot WH2s^32^. Different actin-binding affinities may be required for regulation of actin assembly and dynamics. The sequences surrounding the WH2 motif in various WH2-containing proteins may also contribute to their ability to bind G versus F-actin in an equilibrium fashion, which remains to be investigated. It also remains to be investigated whether the variable sequences in WH2s allow distinct regulatory functions of WH2 domains such as recognizing different proteins other than actin.

The above data have unraveled two critical WH2 motifs, one in PINCH-1, and the other in α-Parvin, which bind to both G-actin and F-actin. The data also suggest a structural basis as to how IPP binds to F-actin via a two-pronged mode. Sequence alignment of PINCH-1 versus PINCH-2 and major Parvin isoforms α-Parvin versus β-Parvin indicated that WH2 motif is present in these proteins (Fig S3F), thus suggesting the conserved actin-binding mode by these IPP isoforms. γ-Parvin, which is predominantly expressed in hematopoietic cells^5^, lacks the N-terminal domain containing WH2-like motif (Supplementary Figure 3F). It remains to be investigated if γ-Parvin confers cell-specific function.

### IPP promotes formation of specific F-actin bundles via WH2s

**Fig. 4:**
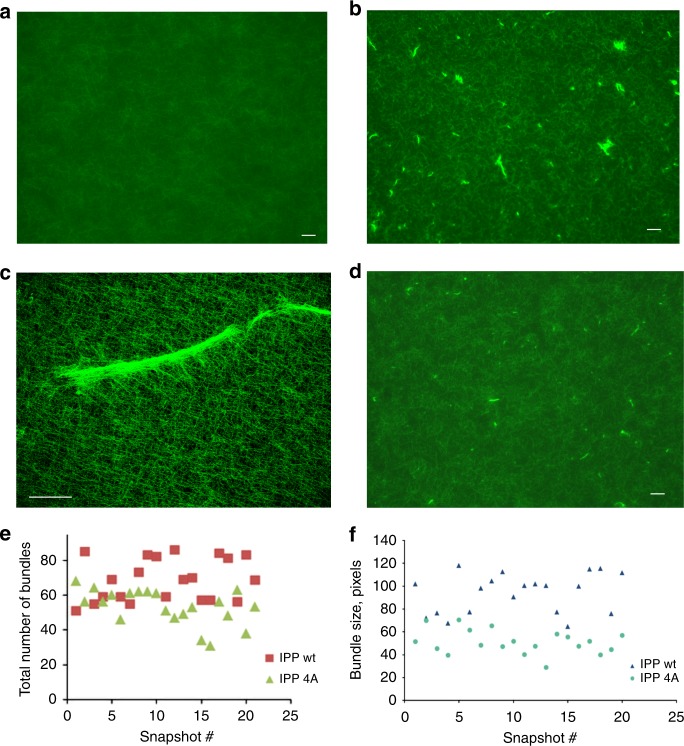
IPP triggers F-actin bundle formation. **a** Selected microscopic image showing the uniform F-actin filament network in the presence of the buffer alone. **b** Selected microscopic image showing F-actin bundles in the presence of IPP. **c** A high resolution image of IPP-induced F-actin bundle detected by confocal microscopy. **d** Selected image showing that IPP-4A mutations led to drastically reduced F-actin bundle formation as compared with (**b**). **e** and **f**. Quantitative comparison on the number (**e**) and size (**f**) of the F-actin bundles induced by IPP and IPP-4A, respectively. Bar = 100 μm except in (**c**) where bar = 50 μm

Next, we wondered how the two-pronged IPP binding to F-actin filaments would regulate actin cytoskeleton assembly and cell adhesion dynamics. The WH2 motif is frequently found in tandem repeats, which can serve as seeding sites for initiating actin polymerization^27,28^. The scenario appears to be different for IPP where a single WH2 motif is located in both PINCH and Parvin, separated by ILK (Fig. 1a). Nevertheless, to examine any potential effect of IPP on actin polymerization, we carried out standard pyrene-based actin polymerization assay (Cytoskeleton, Inc). Supplementary Figure 4A shows that presence of IPP complex even in 1:1 ratio to G-actin did not have significant effect on the actin polymerization. This contrasts to the extreme high potency of Arp2/3 to stimulate actin polymerization (Arp2/3:G-actin = 30 nM:4.3 μM = 1:143) (Supplementary Figure 4A). Since both WH2 motifs of IPP complex interact with F-actin, we then wondered if both WH2 motifs can bind to different F-actin filaments simultaneously and hence induce the filament bundling—a process well-known to generate force/mechanical signal for reorganizing the actin cytoskeleton and thereby triggering dynamic cell adhesion events such as cell spreading and migration^33^. To examine this possibility, we first used a low-speed sedimentation assay—a standard approach for examining F-actin bundles. Supplementary Figure 4B shows that IPP, but not the control buffer, indeed induced formation of F-actin bundles. However, such sedimentation experiments do not reveal morphology of the F-actin bundles and are also less effective for detecting small bundles. We therefore sought to use fluorescent microscopy to visualize and quantify the bundles. In this method, G-actin was polymerized in the absence and presence of IPP, and stained with Alexa488-phalloidin. The sample was then placed on a 22 × 22 mm cover slip, and the area equal to 2.4 × 2 mm was scanned using a wide-field fluorescence microscope, which provides a comprehensive view of F-actin bundles (Supplementary Figure 4C). Figure 4a, b shows representative images of such scans. In the absence of IPP, the F-actin filaments network was uniform (Fig. 4a). However, remarkably, in the presence of IPP, an abundant amount of F-actin bundles was observed (Fig. 4b). A higher resolution image of a representative bundle by confocal microscopy is shown in Fig. 4c. By contrast, in the presence of either PINCH-1 or α-Parvin alone, no F-actin bundles were observed (Supplementary Figure 4D). These data demonstrate that IPP can utilize PINCH-1 and α-Parvin WH2 motifs as two “active hands” to cross-link F-actin filaments and form the specific bundles.

To more definitively evaluate the importance of WH2 motifs in the IPP-induced F-actin bundling, we expressed and purified IPP mutant (IPP-4A) where PINCH-1 LIM5-T WH2 F307, L311, K312, and K313 were mutated into Ala, which resulted in significantly reduced binding of LIM5-T to AP-actin (Fig. 2d) and F-actin (Supplementary Figure 2E). Based on IPP-4A, we also further expressed and purified an IPP mutant (IPP-4A-5A) where α-Parvin WH2 L37, R39, R40, K41, and K42 were mutated to Ala (Parvin-5A) to disrupt the binding of α-Parvin-N to G-actin (Fig. 3b) and F-actin (Supplementary Figure 3C vs. Supplementary Figure 3D). Thus, if we imagine PINCH and Parvin are two arms spatially separated by ILK, and the two WH2s in PINCH and Parvin are two hands that grab/cross-link F-actin filaments, IPP-4A would impair one “hand” of IPP whereas IPP-4A-5A would impair both “hands”. As expected, IPP-4A significantly reduced F-actin bundling (Fig. 4d) as compared with WT IPP (Fig. 4b). Figure 4e, f provides more quantitative illustration, showing that IPP-4A led to the formation of less and smaller sized F-actin bundles than WT IPP. Suppementary Figure 4E shows IPP-4A-5A further reduced the effect as compared with IPP-4A with essentially no F-actin bundles formed. These mutation-based data provide strong supporting evidence that the two WH2s in IPP cooperate to induce F-actin bundling.

### IPP regulates cellular stress fibers and adhesion dynamics

**Fig. 5:**
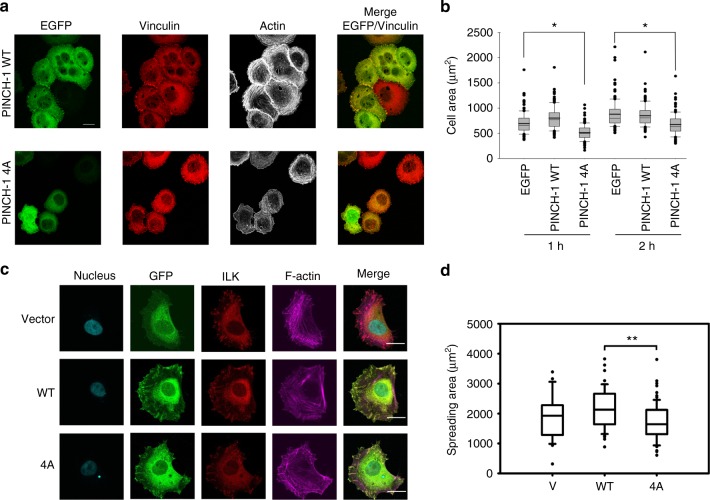
Cellular defects by PINCH-1 4A mutation. **a** Overexpression experiment. HEL cells expressing EGFP-PINCH-1 WT or -1 4A cells were stimulated with 800 nM PMA for 10 min, spread on 20 μg ml^−1^ fibrinogen for 1 h, fixed and stained with Alexa 647-phalloidin to visualize actin and anti-vinculin antibodies to mark FAs. Transfected cells were identified by EGFP fluorescence. Top panels. WT EGFP-PINCH-1 promotes the formation of F-actin stress fibers. By contrast, the stress fibers were substantially disrupted in the cells expressing the mutant PINCH-1-4A (bottom panels). Scale bar, 10 µm. **b** Quantitative analysis of inhibition of cell spreading by PINCH-4A as compared with the WT PINCH-1. Cells were spread on fibrinogen as described in (**a**). The areas of EGFP-positive cells were measured using ImageJ software. *P ≤ 0.001. In total, 150 cells were quantified in each sample. **c** CRISPR/Cas9-based knock-in mutation experiment. HeLa clone (2F5) with frame-shifted PINCH deletion mutant, PINCH-∆C (2F5) was transfected by pEGFP vector (V), pEGFP-PINCH wildtype (WT) or pEGFP-PINCH mutant (4A) and sorted for 2 h spreading on fibronectin coated coverslips (10 µg cm^−2^). Focal adhesions and stress fibers are clearly disrupted by PINCH 4A mutation. Cells were fixed and stained for GFP, ILK, and F-actin. Images were then captured with confocal microscope, under 63x magnification. Scale bar, 20 µm. **d** Quantification of cell spreading with physiological level of PINCH WT/4A expression (P1) in HeLa clone (2F5). Spreading area of 44 cells from each group were quantified with Image Pro plus software based on F-actin staining. Significance was calculated by *t* test. **P < 0.01. The box of the boxplot illustrated the upper and lower quartile for each population (EGFP vector, EGFP-PINCH1-WT and EGFP-PINCH1-4A). Median of spreading area is marked by a horizontal line within the box. The attached whisker indicates the range, and the discrete points (•) are the outliers

To evaluate the importance of the WH2 motifs in IPP-induced F-actin bundling in a cellular context, we first introduced EGFP-PINCH1 or the actin-binding defective EGFP-PINCH1 4 A mutant as well as EGFP alone into HEL cells, a well-established hematopoietic cell model to study integrin function and signaling^34,35^ as regulated by ILK^36^. Transfected HEL cells were stimulated with 800 nM PMA for 10 min to induce integrin activation and plated onto immobilized fibrinogen for 1 or 2 h. Cells were fixed and F-actin visualized with Alexa-647-phalloidin cells expressing EGFP-tagged constructs was identified by EGFP fluorescence, and focal adhesions were identified by vinculin staining. Cell area was determined by measuring 150 cells per each construct. Figure 5a, b shows that while the expression of WT PINCH1 promotes cell spreading, the expression of PINCH1 4A severely impaired it. PINCH1 4A likely competed with endogenous PINCH1, replacing WT IPP with IPP-4A that was defective in F-actin-bundling activity and FA-actin connections, which then led to the disruption of stress fibers and inhibition of cell spreading. To elucidate this in more details and to also eliminate potential complication of endogenous PINCH1 on the PINCH1 4A effect, we created PINCH-1 4A knock-in HeLa cells using the CRISPR/Cas9 technique (Supplementary Figure 5A). The HeLa cells with PINCH-1 4A exhibited dramatically impaired stress fibers and cell spreading (Supplementary Figure 5B, 5C). However, we noticed that the expression level of PINCH-1 4A was very low as compared with that of WT HeLa cells (Supplementary Figure 5E). Such low expression was also seen for a frame-shift-induced PINCH-1 C-terminal deletion mutant (stop codon after F307, named as PINCH-∆C) (Supplementary Figure 5F). We therefore transfected one PINCH-∆C clone 2F5 with EGFP-PINCH constructs and sorted for cells with equal amount of GFP-WT PINCH1 and GFP-PINCH-4A expression (Supplementary Figure 5G). P1 fractions were then applied for 2 h cell spreading on fibronectin-coated coverslips. Figure 5c shows that expression of PINCH-4A led to impaired FAs and stress fibers as compared with that of WT PINCH, although PINCH-4A could still be recruited to FAs (indicated by ILK staining) as compared with the vector control. PINCH-4A also led to the cell spreading defect (Fig. 5d). We also generated α-Parvin knockout HeLa cell line using the CRISPR/Cas9 method. Expression of α-Parvin-5A into the α-Parvin deficient cells exhibited impaired stress fibers and cell spreading as compared with that of WT α-Parvin (Supplementary Figure 6). These data provide strong functional evidence of the importance of PINCH WH2-actin and Parvin WH2-actin interactions in regulating IPP-mediated F-actin assembly and cell adhesion dynamics.

### Mg-ATP bound to ILK regulates IPP-mediated F-actin bundling

**Fig. 6:**
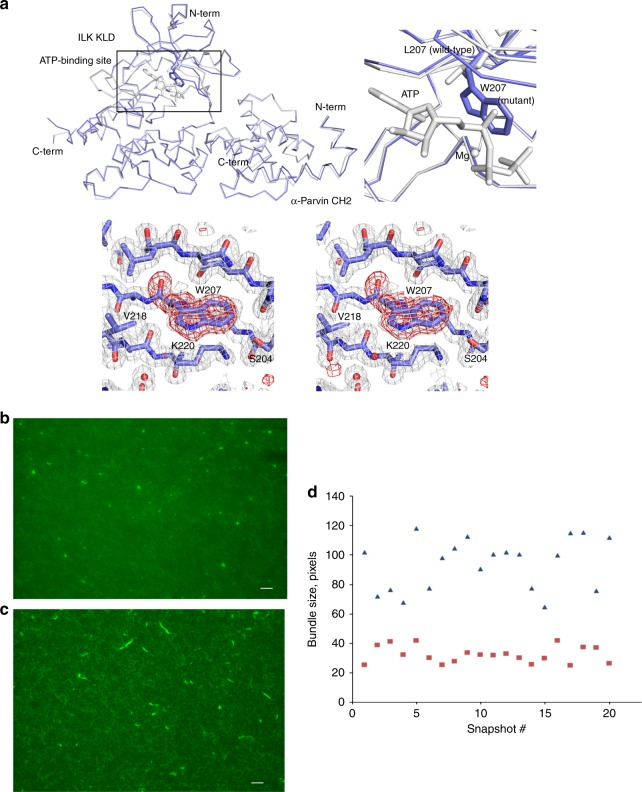
ILK L207W mutation disrupts the F-actin bundling. **a** (Top left) Structural comparison of the ILK KLD bound to α-Parvin CH2 and (colored in gray; PDB ID 3KMW) and its comparison with the ILK mutant form (colored in blue). The superposition of the mutant ILK KLD (267 aligned Cα atoms) to the ATP-bound (PDB ID 3KMW) and -free (PDB ID 3KMU) forms shows overall similarities with root-mean-square deviations of 0.58 Å and 0.47 Å, respectively. A small conformational change is observed in the ATP-binding site of the mutant ILK KLD likely due to mutation or distinct crystal packing. (Top right) Close-up view of the ATP-binding sites of the ILK KLD between ATP-bound wild type (gray) and deficient mutant (blue). Mg, ATP, L207 in the wild type, and W207 in the mutant ILK KLDs are highlighted in ball and stick models. (Bottom) Close-up stereo view of the loss-of-ATP-binding mutation site in the ILK KLD. The 2Fo-Fc electron density map contoured at 1 σ is shown in gray mesh. The Fo–Fc omit map calculated from the mutant structure without the residue (W207), contoured at 3.5 σ, is overlaid (red mesh). Selected residues in the ATP-binding site are labeled. **b** Representative microscopic image showing that IPP L207W impaired F-actin bundle formation (no larger bundles) as compared with the WT IPP in (**c**). Selected microscopic image showing F-actin bundles in the presence of WT IPP. Bar = 100  μm. **d** Quantitative comparison of the F-actin bundle sizes of the randomly selected 20 slides showing the mutation dramatically reduced the F-actin bundle sizes (red squares) as compared with those induced by WT IPP (blue triangles)

**Fig. 7:**
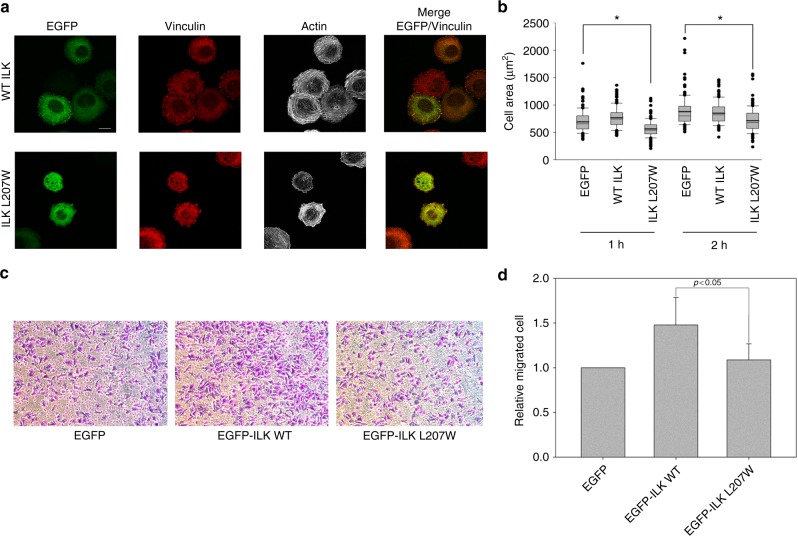
Overexpression of ILK L207W causes defects in cell spreading and migration. **a** HEL cells expressing EGFP-ILK WT or EGFP-ILK L207W were stimulated with 800 nM PMA for 10 min, spread on 20 μg ml^−1^ fibrinogen for 1 h, fixed and stained with Alexa 647-phalloidin to visualize actin and anti-vinculin antibodies to mark focal adhesions. Transfected cells were identified with EGFP fluorescence. (Top panels) WT EGFP-ILK promotes formation of F-actin stress fibers. By contrast, the stress fibers were substantially disrupted in cells expressing the mutant ILK (bottom panels). Scale bar, 10 µm. **b** Quantitative analysis of inhibition of cell spreading by ILK L207W as compared to the WT ILK. The areas of EGFP-positive cells were measured using ImageJ software. *P ≤ 0.001. In total, 150 cells were quantified in each sample. The box of the boxplot illustrated the upper and lower quartile for each population (EGFP vector, EGFP-ILK-WT and EGFP-ILK L207W). Median of spreading area is marked by a horizontal line within the box. The attached whisker indicates the range, and the discrete points (•) are the outliers.  **c** Overexpression of WT ILK but not the L207W mutant in HeLa cells promotes cell migration significantly. Images were captured after 10 h migration, under 10x magnification. **d** Quantitative change of cell migration with WT ILK versus ILK L207W mutant. The relative fold change of migrated cells was calculated from average of five randomly picked fields for each insert. The results were obtained from three independent experiments. Values are given as mean ± SD

**Fig. 8:**
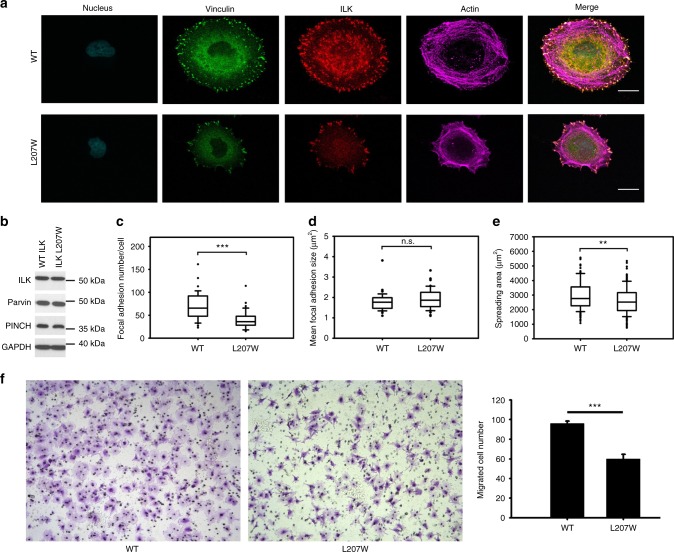
Cellular defects by knock-in mutation (ILK L207W). **a** Comparison of focal adhesions and stress fibers between WT ILK containing HeLa cells and ILK L207W knock-in cells. **b** Western blot of whole-cell lysates from CRISPR generated clones showing that ILK L207W had little effect on the expression of ILK, PINCH, and Parvin. **c**, **d** Quantification of focal adhesion number (**c**) and size (**d**) by WT ILK vs ILK L207W. **e** Cell spreading defects by ILK 207W. The box of the boxplot illustrated the upper and lower quartile for each population (WT ILK and ILK L207W). Median is marked by a horizontal line within the box. The attached whisker indicates the range, and the discrete points (•) are the outliers. **f** WT ILK but not the L207W mutant in HeLa cells promotes cell migration significantly. Images were captured after 10 hours migration, under 10x magnification (left and middle panels). Quantitative change of cell migration by WT ILK versus ILK L207W mutant (right panel). The migrated cell number was calculated from average of five randomly picked fields for each insert. The results were obtained from three independent experiments. Values are given as mean ± S.E.M .Significance was calculated by t test or Mann–Whitney rank sum test based on the normality of data. Scale bar, 20 µm. ***P < 0.001. **P < 0.01

So far, we have shown that ILK specifically recruits PINCH and Parvin into the IPP machinery to trigger dynamic actin bundling. This is a quite significant finding since it provides a basis for understanding how ILK acts as a pseudokinase to promote dynamic FA-actin communication and cytoskeleton reassembly. On the other hand, another major puzzle still remains unresolved, i.e., what is the role of Mg-ATP bound to the ILK pseudoactive site? Mg-ATP is known to be crucial for conventional kinase catalysis but was shown to have little effect either on the overall conformation of ILK or its interaction with Parvin^13,14^—a key step to form the IPP complex (Fig. 1b). Point mutations such as K220M were previously made to disrupt ATP binding to ILK and cause cellular defects^12,37^ but these mutations also partially impair the structural integrity/stability of ILK^14^, making it complicated to assess the role of Mg-ATP. To circumvent this problem, we carefully analyzed the ATP-binding residues in the pseudokinase domain of ILK structure and designed L207W mutation that does not affect the structural integrity but sterically occludes ATP binding to ILK. To validate our design, we first examined the propensity of the substitution by characterizing the recombinant ILK KLD L207W in complex with α-Parvin CH2. The purified ILK KLD L207W mutant complexed with α-Parvin CH2 was expressed in a soluble form similar to that of WT ILK/α-Parvin complex, as judged by its expression and gel filtration profile (Supplementary Figure 7A). Consistently, CD denaturation studies revealed that there was little change in the thermal melting temperature of the mutant ILK (L207W) complex (Tm = 53.9 °C) versus that of the wild-type complex (Tm = 54.2 °C) (Supplementary Figure 7B), demonstrating that the L207W substitution has little effect on the stability of ILK/Parvin complex. To further prove this, we solved the crystal structure of the ILK (L207W)/α-Parvin CH2 complex (Table 2), which showed that the mutant complex exhibits the same interface as the WT complex (Supplementary Figure 7C), yet the substitution of L207 by more bulky side chain of tryptophan in the pseudokinase domain precluded the binding of ATP as well as Mg^2+^ as expected (Fig. 6a). The inability of the ATP binding by the mutant was further validated by florescence-based measurement using MANT-ATP (Supplementary Figure 7D). The mutation clearly abolished the ATP binding as well as Mg^2+^ binding to ILK (Supplementary Figure 7D). We note here that Mg^2+^, while interacting with D339 at the pseudoactive site, is predominantly chelated to ATP (Fig. 6a)^13,14^ explaining why the loss of ATP binding also led to the loss of Mg^2+^ binding. Next, we examined the ability of IPP L207W mutant in affecting the F-actin bundle formation. Strikingly, as compared with WT IPP, loss of Mg-ATP in IPP L207W led to dramatically impaired F-actin bundling (Fig. 6b vs. 6c) with notable decrease in size of the formed F-actin bundles (Fig. 6d). To examine the role of Mg-ATP bound to the ILK pseudoactive site on actin-dependent cellular functions, we expressed EGFP as a control, WT ILK or EGFP-ILK L207W in HEL cells (Supplementary Figure 7E). Transfected HEL cells were treated and examined using the same conditions as we did for PINCH-1 and PINCH-1 4A mutants above. Figure 7a, b shows that the expression of ILK L207W significantly impaired the cell spreading as compared with the WT ILK, which is consistent with the above biochemical data. We also examined how the L207W may affect cell migration that critically depends on actin cytoskeleton change. HeLa cells were transfected with EGFP vector, EGFP-ILK WT and EGFP-ILK L207W, respectively and then subjected to transwell migration assay. Figure 7c, d shows that while expression of WT ILK promotes cell migration, the expression of ILK L207W mutant led to significantly reduced effect. To further elucidate the effect of this mutation in details and to also eliminate potential interference of endogenous ILK on the effect of ILK L207W mutation, we created ILK L207W knock-in mutation using the CRISPR/Cas9 method. Figure 8a shows that ILK L207W led to significantly impaired stress fibers with no change in expression levels of WT ILK vs ILK L207W (Fig. 8b), PINCH1 (Fig. 8b), and Parvin (Fig. 8b). The ILK L207W mutation also reduced FA number (Fig. 8c) while exerting no significant change on FA size (Fig. 8d). Consequently, the mutation further reduced cell spreading (Fig. 8e) and migration (Fig. 8f). It is important to note that like WT IPP, IPP L207W binds potently to F-actin (Supplementary Figure 8A-C). By contrast, IPP L207W has dramatically reduced ability to bundle F-actin as compared with WT IPP (Fig. 6b–d). These results suggest a crucial role of the Mg-ATP binding to ILK in regulating the F-actin bundling, which in turn controls the actin cytoskeleton and cell adhesion dynamics.

**Table 2:** Crystallographic data collection and refinement statistics

*Data collection*	
Space group	P2_1_
Cell dimensions	
*a*, *b*, *c* (Å)	43.71, 117.64, 47.78
α, β, γ (°)	90.00, 114.54, 90.00
Resolution (Å)	50.0–1.8 (1.83–1.80)
Unique reflections	39797 (1954)
*R*_merge_	0.088 (0.463)
*I*/σ*I*	41.78 (7.69)
Completeness (%)	98.9 (95.9)
Redundancy	4.6 (4.2)
*Refinement*	
Resolution (Å)	20.88–1.8 (1.85–1.80)
No. of reflections working set	37699 (2612)
No. of reflections test set	2066 (145)
*R*_work_	0.160 (0.191)
*R*_free_	0.208 (0.240)
No. of atoms	
Protein	3180
Solvent	223
*B*-factors	
Protein	23.88
Solvent	28.41
r.m.s. deviations	
Bond length (Å)	0.021
Bond angles (°)	1.948

Values in parentheses are for highest-resolution shell

## Discussion

In this study, we have attempted to resolve a mechanistic puzzle in cell adhesion: How does ILK transduce signal between integrin-containing FAs and F-actin filaments? We also asked the specific question of why ILK evolved to function as a pseudokinase but still bound to Mg-ATP—the cofactor essential for conventional kinase catalysis? Through a comprehensive set of biochemical, structural, microscopic, and cell biological experiments, we believe we have obtained important clues for addressing these fundamentally important questions.

First, we found that by utilizing its pseudokinase domain to recognize Parvin and its ankyrin repeat domain to recognize PINCH, ILK forms the IPP complex to directly associate with F-actin filaments. This provides the definitive evidence of direct IPP-actin engagement. While IPP was previously widely hypothesized to engage F-actin via Parvin’s CH domains (5, 15, 18), our results showed that it was not the case. Instead, the association occurs in a two-pronged mode involving two previously unrecognized actin-binding WH2 motifs, one from PINCH and the other from Parvin. Remarkably, such two-pronged binding mode allows IPP to trigger the formation of specific F-actin bundles—a process well-known to generate force/mechanical signal for regulating cytoskeleton reassembly. WH2 is known to be a multifunctional actin-binding motif to sequester G-actin as well as promote F-actin assembly (nucleation/elongation/bundling) via distinct mechanisms^28^. In the case of IPP, the two WH2s each located in PINCH and Parvin likely bind G-actin unit of F-actin filaments to promote the F-actin cross-linking and bundling. Since IPP also binds integrin β cytoplasmic tail via the ILK pseudokinase domain without affecting the Parvin binding^13^, our data suggest a dynamic pathway by which ILK acts as the pseudokinase to uniquely engage two WH2s, thereby transducing non-catalytic mechanical force/signal from integrin-containing FAs to F-actin and leading to specific F-actin bundle formation. Such dynamic pathway allows the mechanistic understanding of extensive previous functional data^5,15,18^ that had implicated the essential role of IPP to control cytoskeleton and diverse cytoskeleton-dependent cell adhesive processes such as cell shape change, cell migration, and proliferation. We note that there are several well characterized F-actin-bundling proteins in FAs such as talin, filamin, and actinin, which also physically mediate integrin-FAs-actin linkage^1–4^. However, as we illustrated here, IPP has a distinct tripartite topology where ILK is sandwiched by the WH2-containing PINCH and Parvin, thereby allowing the formation of specific F-actin bundles to fine-tune the stress fiber formation, cell shape, and cell motility. We also note that IPP binds to an array of other FA proteins, some of which may directly or indirectly bind to actin^15^, leading to more complex regulation of actin cytoskeleton dynamics. While such higher level regulation of the actin cytoskeleton remains to be further investigated, the results derived from this study have laid down a foundation for such investigation, signifying an important milestone for understanding ILK-mediated integrin–actin communication and cell adhesion.

Another major finding of this study was that the IPP WH2-mediated F-actin bundling is highly sensitized to Mg-ATP bound to the pseudoactive site of ILK. This was strongly demonstrated by our mutagenesis data at both biochemical and cellular levels. A large portion of pseudokinases binds ATP with largely unknown functional bases^38^. Previous studies have suggested that ATP might structurally stabilize a pseudokinase or induce its conformational change, thereby altering the binding to and/or activity of its partner^38^. A definitive example is that ATP induces a conformational change of a pseudokinase STRAD-α (no Mg^2+^ involvement) to activate downstream binding target LKB kinase^39^. However, Mg-ATP has little effect on the overall conformation of ILK in complex with Parvin^13,14^. Thus, the Mg-ATP binding to ILK may adopt a totally different mechanism to regulate the pseudokinase function, which remains to be determined. Nevertheless, our data at least unraveled an important phenomenon where the ILK bound Mg-ATP regulates the F-actin-filament assembly, which in turn controls the actin cytoskeleton and diverse cytoskeleton-dependent cell adhesive and signaling events.

In summary, we believe that our study marks a significant advance for understanding the ILK function and many ILK-dependent physiological/pathological processes. Our data also shed light upon the ability of pseudokinases to evolve as distinct non-catalytic signal transducers. Since ILK or IPP is highly upregulated in multiple diseases, such as cancer, diabetes, and heart failure^5,7,15,17,18^, our findings also open the door for developing unique therapeutic inhibitors of ILK, not as a kinase, but as a pseudokinase, by targeting at its Mg-ATP site or its binding to other proteins.

## Methods

### DNA constructs, protein expression, and purification

The gene of PINCH1 LIM5-T (residues 248–325) was sublconed into pGEX-4T-1 (Pharmacia) vector using *BamHI* and *XhoI* sites. For expression of LIM5-T cultures were grown at 37 °C to OD_600_≈0.6 and induced at room temperature overnight (18–20 h) with 0.2 mM IPTG. LIM5-T was purified using a standard protocol for purification of GST-fused constructs (described in details in ref. ^40^). GST tag was cleaved with Thrombin and the digestion mixture was purified on Sephadex 75 resin (Amersham Biosciences).

Full-length thymosin β4 was subcloned into PET15b vector (Novagen) using *NdeI* and *BamHI* sites and transformed into *E. coli* Bl21(DE3) cells for expression. Cell cultures were grown at 37 °C to OD600≈0.6, induced with 1 mM IPTG at grown at 37 °C for 4 h. Cell lysates were applied to Ni-NTA resin (Novagen) and eluted in presence of 500 mM imidazol. Elution fractions were pooled, buffer exchanged into imidazole free buffer. His tag was cleaved with Thrombin protease and then additionally purified with Superdex Peptide 10/300 GL column (Amersham Pharmacia) in low-salt buffer (4 mM NaH_2_PO_4_, 0.2 mM CaCl_2_, 0.2 mM ATP, 0.5 mM DTT, pH 6.5). Protein was quantified using BCA protein assay (Pierce).

The recombinant heterotrimer complex (IPP complex) consisting of ILK, PINCH-1, and α-Parvin was generated by a bacterial tricistronic coexpression system. Briefly, genes encoding the full-length human ILK (residues 1–452), human PINCH-1 (residues 1–325), and human α-Parvin (residues 1–372) were PCR-amplified and subcloned into a polycistronic coexpression vector pST39^41^ according to the inventor’s protocol. Two surface cysteine residues of ILK were substituted by serine residues (C346S; C422S) to improve the protein solubility of ILK, as previously demonstrated^13^, and designated as the IPP-WT. The tricistronic coexpression construct contains a hexahistidine-tag sequence followed by a thrombin cleavage site prior to the sequence of PINCH-1 for conventional affinity purification. The hexahistidine-tagged IPP complex was coexpressed in *E. coli* BL21(DE3) pLysS and purified from the bacterial cell lysate by Ni-affinity chromatography column followed by HiLoad 16/60 Superdex 200 gel filtration and HiTrap SP cation-exchange chromatography columns (all from GE Healthcare). The mutant proteins (IPP L207W, IPP-4A, and IPP-4A5A) of the IPP complex were generated by QuikChange Site-Directed Mutagenesis kit (Agilent), and were expressed and purified as for the IPP-WT. For GST-fused various fragments of human α-Parvin, each gene of the N-terminal domain (residues 1–89), or its mutant 5A (1–91) or the full-length (residues 1–372) of α-Parvin was subcloned into pGEX4T1 vector (GE Healthcare). These GST-fused α-Parvin proteins were expressed in *E. coli* and affinity-purified by glutathione-Sepharose 4B affinity column (GE Healthcare). For hexahistidine-tagged fragments of α-Parvin, each gene of the N-terminal domain (residues 1–91) or CH1–CH2 (residues 89–372) or NCH1-CH2 (residues 69–372) was subcloned into a pET15b vector (Novagen). These hexahistidine-tagged various fragments of α-Parvin were expressed in *E. coli* and purified by Ni-affinity chromatography followed by size exclusion chromatography column of HiLoad 16/60 Superdex 200 (GE Healthcare). For the MBP-fused PINCH1 protein, the gene of human PINCH1 (residues 1–325) was subcloned into a pMALc2x vector (NEB). The MBP-fused PINCH1 was expressed in *E. coli* and purified by amylose resin (NEB) affinity chromatography followed by HiLoad 16/60 Superdex 200 chromatography column. The full-length ILK bound to MBP-fused PINCH1-LIM1-2 protein and the ILK KLD wild type (WT) bound to α-Parvin CH2 were generated, as previously demonstrated^13^. The mutagenesis for the loss-of-actin binding mutants of PINCH and Parvin fragments was performed as described above. All the expression plasmid constructs were verified by DNA sequencing analysis.

Nonpolymerizable recombinant AP-actin was prepared using the recombinant baculovirus from the Trybus lab (University of Vermont), as previously demonstrated^26^. The protein yield of the recombinant AP-actin was about 10 mg of the protein L^−1^ of insect cells.

Primers used for the above expression constructs and cell lines are listed in Supplementary Table 1.

### NMR sample preparation

To prepare ^15^N and/or ^13^C and/or ^2^H labeled PINCH1 LIM5-T for NMR experiments, *E. coli* BL21 (DE3) cells were grown in M9 minimal medium containing 1.1 g l^−1 15^N-NH4Cl and/or 3.3 g l^−1 13^C-glucose, and/or 90% D_2_O. The uniformly labeled LIM5-T protein was purified as described above. The hexahistidine-tagged N-terminal domain proteins (residues 1–91 both for WT and 5 A mutant) and CH1–CH2 domain (residues 69–372) of α-Parvin were expressed in M9 minimal medium supplemented with ^15^N ammonium chloride as described above and purified by standard affinity and size exclusion chromatography protocol, as described above. The N-terminal hexahistidine-tag was removed by thrombin digestion, and the uniformly labeled α-Parvin proteins were further purified by Resource-Q chromatography column (GE Healthcare). These ^15^N-labeled proteins were prepared in a buffer consisting of 5 mM Tris, pH 7, 0.2 mM CaCl_2_, 0.5 mM DTT, 0.2 mM ATP, and 10 mM NaCl, and 10% D_2_O was supplemented when titration experiments with AP G-actin were performed.

To prepare thymosin-β4/G-actin complex, a 10 μM bovine cardiac muscle actin (Cytoskeleton, Inc) solution was prepared by dissolving of corresponding amount of lyophilized actin powder in the low-salt NMR buffer: 4 mM Na_2_HPO_4_, 0.2 mM CaCl_2_, 0.2 mM ATP, 0.5 mM TCEP, pH 6.5. Thymosin-β4 in the same buffer was added in an adequate amount resulting in a 1:1 molar ratio with actin. The excess of free thymosin-β4 and remaining actin oligomers were removed by gel filtration using Superdex 75 10/300 GL (Amersham Biosciences) column equilibrated in G-actin buffer. Fractions containing thymosin-β4-G-actin complex were pooled together and concentrated with Vivaspin concentrators 10 K MWCO (Vivascience) to a final concentration of 0.2–0.3 mM.

### NMR spectroscopy

The structure of LIM5-T (9 kDa) was determined by standard triple resonance experiments^42^ on Bruker Avance 600 MHz spectrometer. NMR sample of 1 mM ^1^H, ^15^N, and ^13^C-labeled LIM5-T was prepared in 25 mM Na_2_HPO_4_, 20 mM NaCl, 0.1 mM TCEP, pH 6.5 buffer, and a set of through-bond triple-resonance experiments, including HNCO, HNCA, CBCANH, CBCACONH, C(CO)NH, and H(CCO)NH, was used for backbone resonance assignment^42^. The side chain assignments were made by analyzing HCCH-TOCSY data. For semi-automatic NOE analysis, we used Pipp and Stapp software^43^. Backbone ϕ, ψ torsion angle restraints were derived from a database analysis of backbone (N, HN, Cα^,^ Cβ, C’, Hα) chemical shifts using the program TALOS+^44^. ^1^H–^1^H distance restraints were derived from 3D ^15^N-separated NOESY and 3D ^13^C-separated NOESY respectively involving sequential and tertiary NOEs for LIM5-T^43^. The NOE analyses was done iteratively with structure calculations. The structure was calculated with X-PLOR-NIH program^45^ (Table 1). Molprobity analysis at the PDB site (https://www.rscb.org) shows that 97.9% of the residues in the calculated structures are in allowed region and 2.1% are in disallowed region. Structure quality was evaluated with program Procheck-NMR^46^. All NMR data were processed and analyzed by the program NMRPipe^47^.

### X-ray crystallography

The ATP-binding deficient mutant (L207W) of human ILK KLD was bacterially coexpressed with human α-Parvin CH2 and purified according to the previously described protocol for the ILK KLD wild type^13^. Two surface cysteine residues of ILK KLD were substituted by serine residues (C346S; C422S), as previously documented. Crystals of ILK KLD (L207W) bound to α-Parvin CH2 were grown at 4 °C by the hanging drop vapor diffusion method using the reservoir containing 16% polyethylene glycol 3350, 0.1 M Bis-Tris Propane, pH 7.4, and 5% 1-propanol. Crystals were transferred to 20% glycerol supplemented in the mother liquor and flash-frozen in liquid nitrogen. X-ray diffraction data were collected at 100 K on beamline 19-BM (λ = 0.979 Å) at the Advanced Photon Source. The diffraction data were processed by the HKL-3000 program^48^. Phases were obtained by molecular replacement method with MOLREP^49^ using the coordinates of the wild-type ILK KLD bound to α-parvin CH2 (PDB ID 3KMW) without solvent and ligands. The initial coordinates were subjected to a rigid-body protocol using the program REFMAC^50^. The amino acid substitution of Leu207 by Trp207 in the mutant structure of ILK KLD was verified by computing density maps of |2*F*_o_–*F*_c_| and |*F*_o_–*F*_c_| with an aid of the modeling program COOT^51^. The mutant structure was refined to a resolution at 1.8 Å using the program REFMAC^50^. The structure figures were drawn using the program PyMol (www.pymol.org).

### Quantitative fluorescence-based ATP-binding experiment

The binding of fluorescence nucleotide analog MANT (N-methylanthraniloyl)-ATP to the ILK proteins was investigated by measuring a fluorescence energy transfer signal from tryptophan of the protein to MANT-ATP. The bacterially expressed and purified ILK KLD (either WT or L207W mutant) in complex with α-Parvin CH2 was prepared at a final concentration of 1 μM in the solution (at a final volume of 80 μL) consisting of 20 mM HEPES, pH 7.5, 150 mM NaCl, 5 mM either MgCl_2_ or no cation, and 4.8% glycerol in a 96-well black flat-bottom microplate (Greiner Bio-One). MANT-ATP (Life Technologies) was titrated in the reaction solution. The reaction mixtures were incubated at room temperature at 150 rpm for 10 min, and the fluorescence intensity was measured on a 2300 EnSpire Multimode Plate Reader (PerkinElmer) with excitation (280 nm) and emission (430 nm) wavelengths. The fluorescence signal was normalized by subtracting the background fluorescence of the MANT-ATP buffer, and plotted as a function of the concentration of MANT-ATP. The binding constant of MANT-ATP to ILK KLD was estimated by one-site total binding fit model using the program GraphPad Prism. The binding results were obtained in two-independent experiments performed in duplicate.

### CD-based thermal shift assay

CD measurements were performed with AVIV-215 equipped with a computer controller Peltier sample holder. All measurements were taken in a 1 mm pathlength cell with sample concentration at 0.2 mg mL^−1^. The thermal stability of wild type and L207W mutant was monitored at 222 nm by heating samples at a rate of 60 °C h^−1^ over the range of 10–70 °C. The ellipticity was recorded at 0.5 °C intervals with time constant of 16 s. The data were analyzed by Origin 2017.

### F-actin co-sedimentation assay

The F-actin-binding experiments were performed according to the manufacturer’s protocol (Cytoskeleton, Inc.). Briefly, rabbit skeletal muscle lyophilized actin (Cytoskeleton, Inc) was dissolved in G-buffer (5 mM Tris-HCl, pH 8.0), 0.2 mM CaCl_2_, 0.2 mM ATP, 0.5 mM DTT, 1 mM NaN_3_) to the concentration of ~8–10 μM, incubated on ice for 1 h and then centrifuged at 150,000xg for 1 h at 4 ^o^C. Test proteins were precleared in the same way. Then G-actin was polymerized by addition of salts (50 mM KCl, 2 mM MgCl_2_, 1 mM ATP) and the actin solution was incubated at room temperature for 1 h. The test proteins were added in the F-actin buffer only (either with 5% glycerol or no glycerol) and the F-actin stock, and then incubated at room temperature for 30 min. The reaction mixtures were centrifuged at 150,000xg at 4 °C for 1 h in Optima TLX Ultracentrifuge (Beckman), and the supernatants and the pellets were carefully fractionated. Both the pellets and the supernatants were analyzed by SDS-PAGE, and the gels were stained by Coomassie Brilliant. The densitometric quantification of the bands in the pellets was performed by ImageJ software (NIH). Normalized binding data with blank subtraction were obtained by dividing the values of pelleted fractions of a protein by the maximum value of the protein in the pellet at saturation, and plotted as a function of the protein concentration. Curve fitting and K_D_ calculation were performed by GraphPad Prism (GraphPad Software, Inc).

### Pyrenyl-actin polymerization

We used ~3 μM G-actin (~5% pyrenyl-labeled) solution and observed its polymerization upon addition of salts (50 mM KCl, 2 mM MgCl_2_, 1 mM ATP) in the absence and in the presence of ~3 μM IPP complex. IPP was always premixed with salt before addition to actin solution. Arp2/3 and VCA (WASP VCA domain) were purchased from Cytoskeleton, Inc, reconstituted according to the manufacturers’ instructions. Final sample concentration of Arp2/3 and VCA were 20–30 nM and 70 nM, correspondingly. Polymerization rate was monitored in situ by measuring fluorescence intensity with excitation and emission wavelengths 365 nm and 407 nm, respectively, using 2300 EnSpire Multimode Plate Reader.

### Fluorescence microscopy of F-actin filaments

Rabbit skeletal muscle lyophilized actin (Cytoskeleton, Inc) was dissolved in G-buffer (5 mM Tris-HCl, pH 8.0), 0.2 mM CaCl_2_, 0.2 mM ATP, 0.5 mM DTT, 1 mM NaN_3_) to the concentration of ~8 μM, incubated on ice for 1 h and injected into Superdex 75 10/300 GL (Amersham Biosciences) gel filtration column. Monomer fractions were collected and used for the experiment. Usually, the fractions contained G-actin concentration of 3–5 μM. Then we polymerized 1 μM G-actin by addition of salts (50 mM KCl, 2 mM MgCl_2_, 1 mM ATP) in the presence of IPP buffer or IPP protein (1:1 molar ratio) and the filaments were labeled with ~1 μM Alexa 488-phalloidin (Sigma). Prior to the visualization, 1 μl of sample was added to 9 μL of F-buffer (G-buffer above plus salts) and applied to a cover slip coated with poly-L-lysine (0.01%). Widefield images were acquired using a Leica DM5000B upright fluorescence microscope (Leica Microsystems, GmbH, Wetzlar, Germany), equipped with a Retiga SRV camera and QCapture Pro software (QImaging, Surrey, BC Canada). Confocal images were acquired using a Leica TCS-SP5 II upright confocal microscope (Leica Microsystems, GmbH, Wetzlar, Germany).

### Cell culture

HeLa cells (ATCC CCL-2) and HeLa knock-in/knockout cells were maintained in Dulbecco’s Modified Eagle’s Medium supplemented with 10% fetal bovine serum. HEL cells (ATCC TIB-180) were maintained in RPMI-1640 Medium supplemented with 10% fetal bovine serum. All cells were kept at 37 °C in incubator with 5% CO_2_.

### Generation of knock-in cells

The genome of HeLa cells was edited by CRISPR/Cas9 system^52^ to get knock-in cell clones. CHOPCHOP web tool^53^ was used for guideRNA design. Four guideRNAs around ILK target site and two guideRNAs around PINCH target site were selected and synthesized by in vitro transcription (GeneArt Precision gRNA synthesis kit, Fisher), then applied for genomic cleavage test (GeneArt Genomic Cleavage Detection kit, Fisher). The optimal target sequence was tested to be CC CTA CCT GTC CTG CAG CTA TGG (for ILK L207W) and GT GCT ATG AGA AAT TTC CAT (for PINCH 4 A). Single stranded DNA donor was designed asymmetrically^52^) and synthesized with two ends modified by phosphothioate bonds (Supplementary Table 1).

Early passages of HeLa cells (ATCC CCL-2) were cotransfected with wild-type Cas9 mRNA(Trilink), guideRNA, and single-stranded DNA donor (IDT) by Lipofectamine MessengerMAX (Fisher) at 70% confluency in six-well plates. Forty-eight hours after transfection, cells were dissociated (Cell dissociation buffer, Fisher) and applied for single cell sorting (FACSAriaII, performed by Cleveland Clinic Flow Cytometry Core) into 96-well plates. Cells were allowed to grow for 10–14 days after sorting and single-cell clones were picked for expansion and sequencing. Identified ILK L207W and PINCH-1 4 A knock-in clones were subjected to off-target sites sequencing which was predicted by CHOPCHOP web tool^53^. No off-target was detected.

### Generation of α-Parvin knockout cells

The genome of HeLa cells was edited by CRISPR/Cas9 system to generate knockout cell clones. Optimal target sequence was tested to be TC ATC TTT CTT GCG GGA CGG GGG (PARVA KO). Early passages of HeLa cells (ATCC CCL-2) were cotransfected with wild-type Cas9 mRNA(Trilink) and guideRNA by Lipofectamine MessengerMax (Fisher) at 70% confluency in six-well plate. Forty-eight hours after transfection, cells were harvested by trypsin and seeded into 96-well plate. Cells were allowed to grow for 2 weeks and single-cell clones were picked for expansion and western blot verification.

### Mammalian plasmids constructs and transfections

PINCH-1, PINCH-4A, α-Parvin, α-Parvin 5A, ILK, and ILK L207W constructs were subcloned into pEGFP-C2 vector and purified by QIAprep Spin Miniprep Kit (Qiagen) and PureYield Plasmid Midiprep System (Promega). Transient nucleofections of HEL cells were performed using nucleofection kit V from Lonza (Walkersville, MD), according to manufacturer instructions and program X-005 with 3 µg of DNA per sample. Transient transfection of HeLa cells were performed using PEI transfection reagent.

Primers of these mammalian constructs and cell lines are listed in Supplementary Table 1.

### Western blotting

Cells were lysed in lysis buffer (50 mM Tris-HCl, pH 6.8, 1% SDS) and applied for protein quantification (Pierce BCA protein assay kit, Fisher). Lysates were diluted in Laemmli buffer containing 62.5 mM Tris-HCl, pH 7.4, 2% SDS, 5% 2-mercaptoethanol, and 10% glycerol to the same concentration and subjected to SDS-PAGE. Proteins were then transferred to PVDF and probed with primary antibodies (anti-GFP, Cell Signaling; anti-ILK, Abcam; anti-GAPDH, Cell Signaling; anti-PINCH, BD Bioscience; anti-Parvin, Cell Signaling; anti-actin, Cell Signaling), which was followed by HRP-conjugated secondary antibody (anti-mouse HRP, Cell Signaling; anti-rabbit HRP, Cell Signaling). All antibodies and their dilution in the study are listed in Supplementary Table 1, uncropped western blots are provided in Supplementary Figures 9–14.

### Cell sorting

HeLa 2F5 cells (1 bp del, leading to PINCH C-terminal deletion with decreased expression level) transfected with EGFP-tagged constructs were harvested by Enzyme Free Cell Dissociation Buffer (Fisher) after 24 h and subjected to cell sorting (BD FACSAriaII, Cleveland Clinic). P1 fraction was collected and recovered in medium overnight in 37 °C incubator. Recovered cells were then subjected to cell spreading and Western blot.

### Cell spreading assays

HEL cells transfected with EGFP-tagged constructs were stimulated with 800 nM PMA for 5 min and incubated with immobilized fibrinogen in duplicate, for 1 h or 2 h at 37 °C in serum-free medium. After extensive washing with PBS, the adherent cells were fixed with 4% PFA and stained antivinculin antibody (hVIN-1 clone, Sigma-Aldrich, St. Louis, MO), followed by secondary antimouse goat F(ab) fragment antibody Alexa 568-conjugated (Thermo Fisher Scientific) and with Alexa 647-phalloidin (Thermo Fisher Scientific). The cells were visualized with 40x and 63X objectives using a Leica TCS-NT laser scanning confocal microscope and the cell area was measured for 150 cells with ImageJ software.

HeLa cells after CRISPR or cell sorting were seeded to fibronectin-coated coverslips (10 µg/cm^2^) for 2 h spreading at 37 °C. Adherent cells were fixed with 4% PFA and stained with anti-vinculin antibody (Sigma) or anti-GFP antibody (Abcam) and anti-ILK antibody (abcam) followed by goat anti-mouse antibody Alexa 488-conjugated (abcam) or goat anti-chicken antibody Alexa 488-conjugated (Abcam) and goat anti-rabbit Alexa 568-conjugated (abcam). Coverslips were then mounted with Prolong Gold Antifade Reagent with DAPI (Fisher) overnight and visualized with Leica TCS-SP5 II upright confocal microscope (Leica Microsystems, GmbH, Wetzlar, Germany).

### Cell migration assays

HeLa cells transfected with pEGFP-c2 vector, pEGFP-ILK WT and pEGFP-ILK L207W were serum starved overnight after 30 h transfection and harvested for migration assay. Similarly, WT ILK and L207W knock-in cell clones were serum starved overnight before migration assay. Harvested HeLa cells were resuspended in serum-free medium at the concentration of 7.5 × 104 cells ml^−1^, and 200 µl was added to cell culture inserts (8 µm pore size, 24-well, Falcon) coated with 10 µg ml^−1^ fibronectin (EMD Millipore). In total, 800 µl medium with 10% FBS was added outside each insert as chemoattractant. The 24-well plates were incubated for 10 h at 37 ^o^C in an incubator with 5% CO_2_ and then inserts were fixed and stained by methanol and crystal violet. The noninvasive cells on the upper surface of the membrane were removed by wiping with swabs and then inserts were imaged under inverted brightfield microscope. Typically, five views were counted and averaged for each insert. All experiments were performed for three times.

### Statistical analysis

Two different tests were applied based on data distribution to show the significance: *t* test and Mann–Whitney rank sum test. After data collection, all raw data went through normality test and equal variance test in SigmaPlot 10.0 software. Then, only the ones passed both tests were applied for *t* test. Otherwise, data were compared by Mann–Whitney rank sum test. Differences were considered to be significant when *P* < 0.05.

## Electronic supplementary material

Supplementary Information

## Data Availability

The atomic coordinates of PINCH LIM5-T and ILK KLD (L207W)/Parvin CH2 complex have been deposited in the Protein Data Bank (accession codes: 6 MIF and 6 MIB, respectively). All other data supporting the findings of this study are available from the corresponding author qinj@ccf.org upon reasonable request.
