# Supplementary material for: Non-catalytic signaling by pseudokinase ILK for regulating cell adhesion
Source: Nat Commun. 2018 Oct 26;9:4465. doi: 10.1038/s41467-018-06906-7 (PMC6203859; doi:10.1038/s41467-018-06906-7)
Supplement: Supplementary file 1 — Supplementary Information [file 41467_2018_6906_MOESM1_ESM.pdf]

**Supplementary Information For**

# Non-catalytic signaling by pseudokinase ILK for regulating cell adhesion

Julia Vaynberg et al.

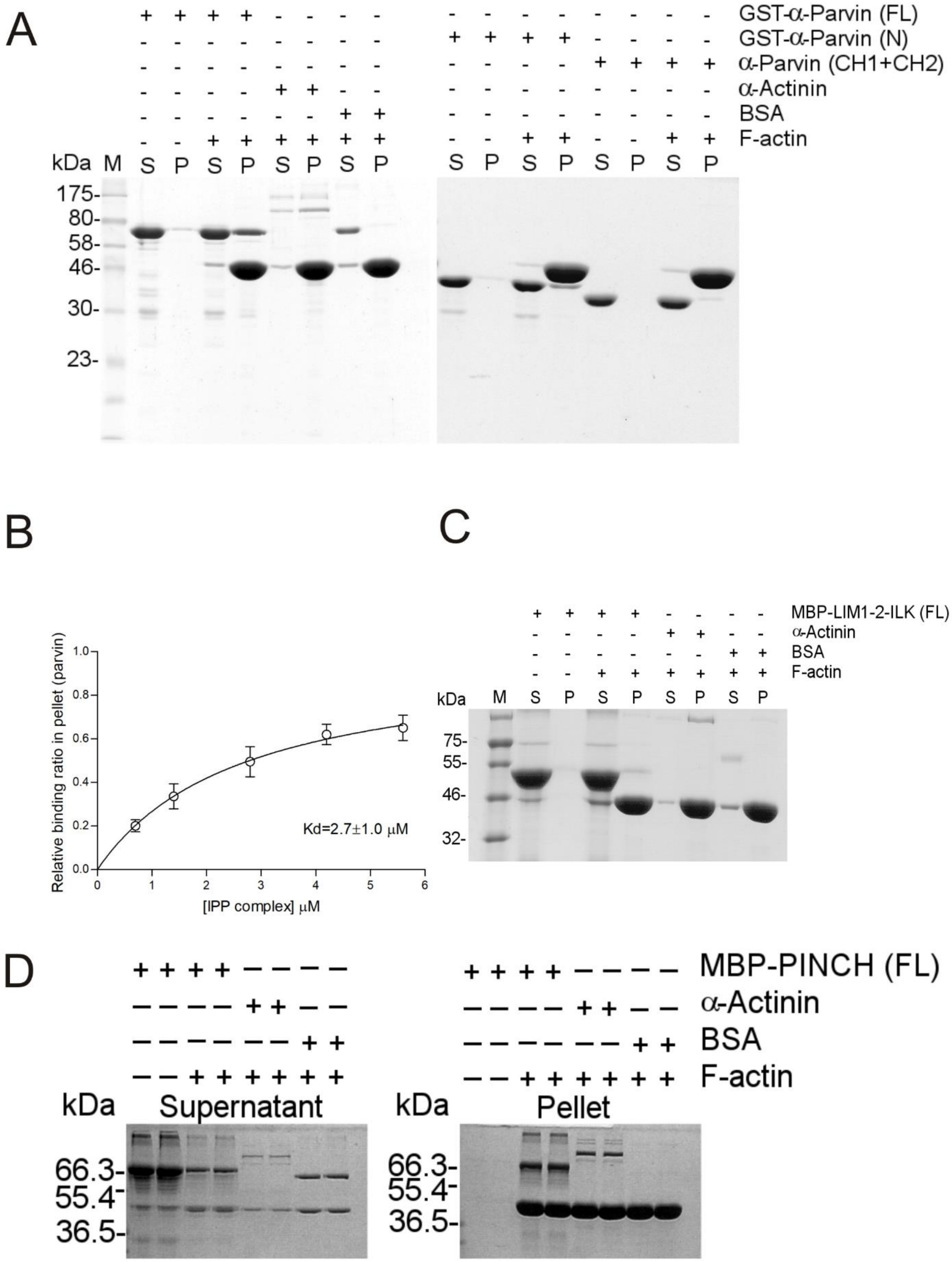
A
GST-α-Parvin (FL)
GST-α-Parvin (N)
α-Parvin (CH1+CH2)
α-Actinin
BSA
F-actin
kDa M S P S P S P S P S P S P S P S P
175-
80-
58-
46-
30-
23-
B
Relative binding ratio in pellet (parvin)
[IPP complex] μM
Kd=2.7±1.0 μM
C
MBP-LIM1-2-ILK (FL)
α-Actinin
BSA
F-actin
kDa M S P S P S P S P
75-
55-
46-
32-
D
MBP-PINCH (FL)
α-Actinin
BSA
F-actin
kDa Supernatant
kDa Pellet
66.3-
55.4-
36.5-

**Supplementary Figure 1**. **Determination of IPP subunits responsible for binding to actin using F-actin co-sedimentation assay**. (**A**) Investigation of various fragments of α-Parvin for binding to F-actin. The full-length (FL) α-Parvin binds F-actin. α-Parvin CH1-CH2 did not show significant binding to F-actin, whereas the N-terminal domain (N) binds potently to F-actin. (**B**) Binding affinity ($K_D$ = 2.7±1.0 μM) of IPP to F-actin as derived from the binding fraction of α-Parvin in pellet as a function of the IPP concentration (three independent experiments). The $K_D$s estimated using the binding fraction of ILK and PINCH are similar (2.2±0.6 μM and 4.4±3.9 μM, respectively). The relative amounts of each protein in each fraction (pellet) were estimated by scanning densitometry of the Coomassie Blue-stained SDS-gels, background-normalized, and plotted as a function of the concentration of IPP. The maximum amounts of the binding of each protein to F-actin (Bmax) were estimated by one-site binding analysis with Graphpad Prism. The relative binding ratio of each protein from three independent experiments was obtained by dividing each normalized densitometry by each Bmax. (**C**). Full length ILK has little binding to F-actin. Note that the full length ILK was prepared in the complex form with PINCH1 LIM1-2 fused to maltose binding protein (MBP) since ILK alone is not soluble. (**D**) Full length PINCH-1 binds to F-actin. PINCH-1 was fused to MBP.

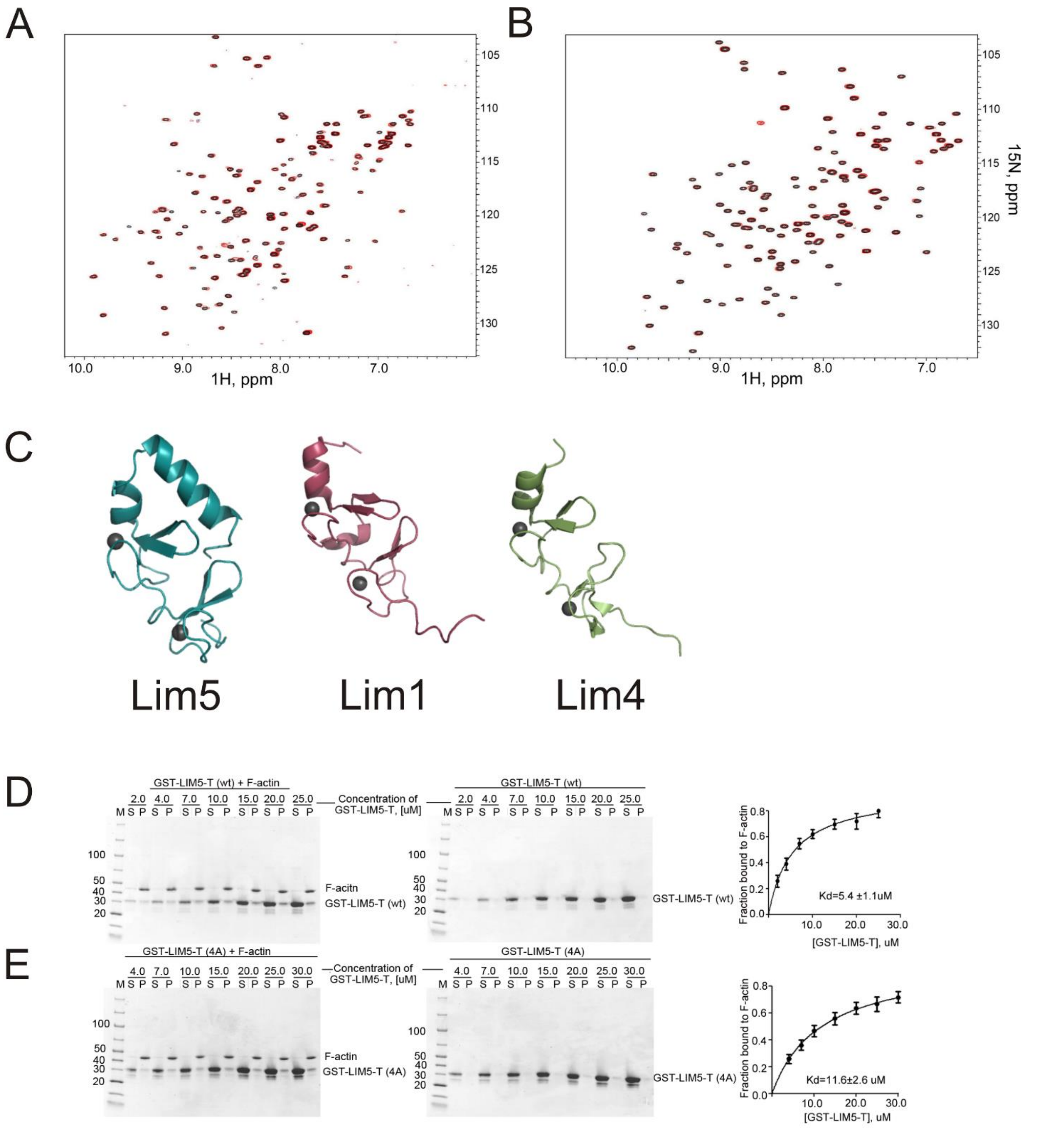


**Supplementary Figure 2. Analysis of PINCH-1 LIM domains**. (**A**) 0.1 mM $^{1}$H-$^{15}$N HSQC of PINCH-1 LIM1-2 in the absence and presence of 0.2 mM AP-actin showing that PINCH-1 LIM1-2 has little binding to G-actin. (**B**) 0.1 mM $^{1}$H-$^{15}$N HSQC of PINCH-1 LIM3-4 in the absence and presence of 0.2 mM AP-actin showing that PINCH-1 LIM3-4 has little binding to G-actin. (**C**). Structural comparison of PINCH-1 LIM1, LIM4, and LIM5 showing the similar fold. RMSD is 3.5 Å between 49 equivalent C$\alpha$ atoms of LIM5 residues V251-E305 and Lim1 A192-Q246 and 1.5 Å between 52 equivalent C$\alpha$ atoms of LIM5 residues V250-E304 and LIM4 I192-Q246, respectively. (**D**) GST-LIM5-T and (**E**) GST-LIM-T 4A binding to F-actin by high-speed F-actin co-sedimentation experiments. Samples of protein with F-actin at different concentrations and control samples without F actin were processed and all pellets with and without F actin were run on the same gel for quantification

by ImageJ software. Fraction of bound protein was derived from [Intensity of pellet band (protein + F-actin) - Intensity of pellet band (protein)] $Bmax^{-1}$. Bmax was obtained from the projected saturation point in Graphpad Prism that estimated $K_D$ by one-site total binding fit model. Pellets and supernatants from samples of GST-LIM5-T (**D**) and GST-LIM-T 4A (**E**) are shown. Three independent experiments were carried out, and the averaged results are shown indicating that the 4A mutation (**E**) significantly reduced the LIM5-T binding to F-actin.

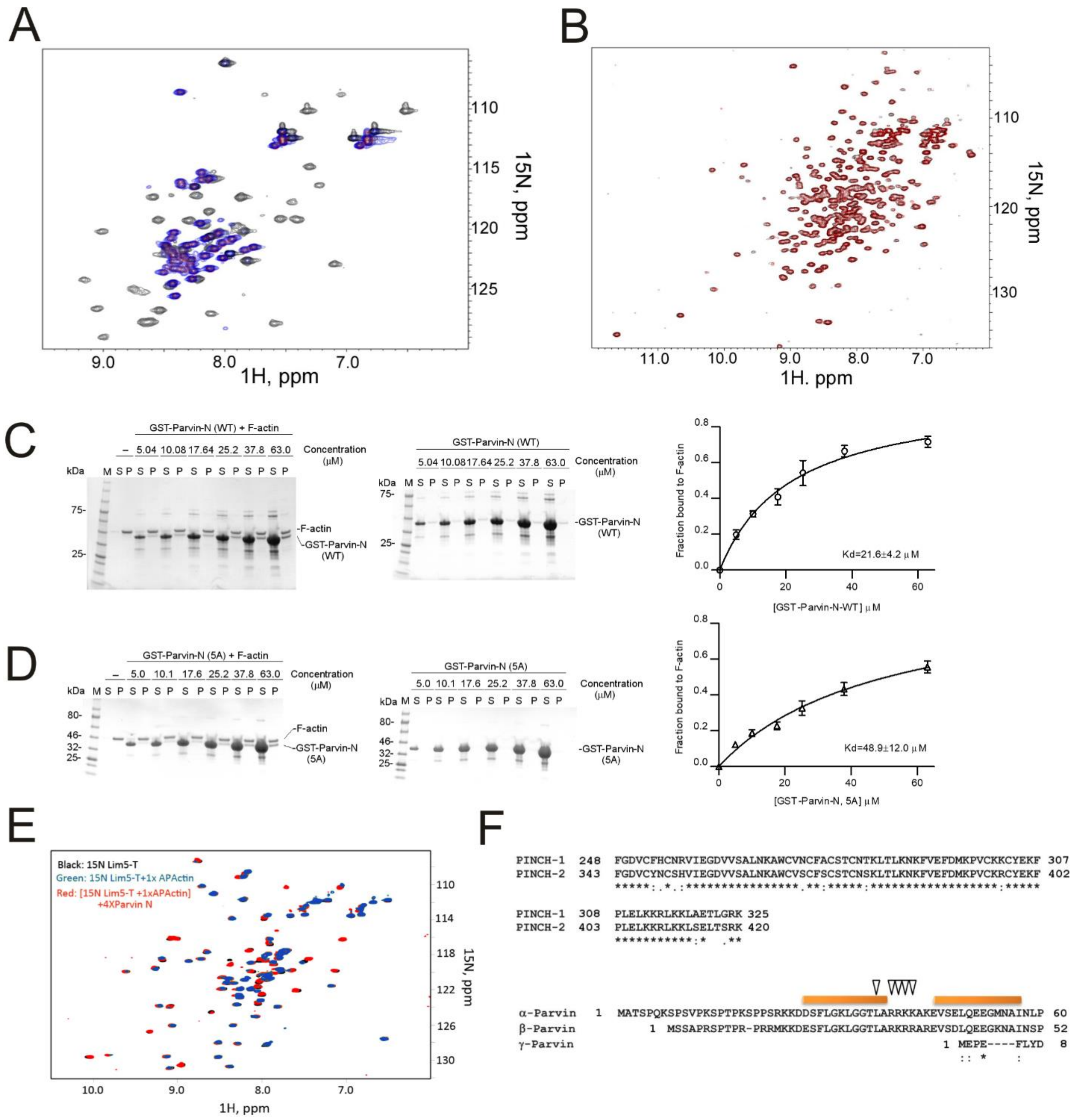


**Supplementary Figure 3. Analysis of actin binding site in PINCH and Parvin**. (**A**) 0.1 mM $^{1}$H-$^{15}$N HSQC of thymosin-β4 in the absence (blue) and presence of 0.1 mM G-actin (black) and presence of 0.1 mM G-actin and 0.3 mM LIM5-T (red). Notice that the widely dispersed peaks of thymosin-β4 bound to AP-actin (black), e.g., peaks in $^{1}$H 8.5-9.2 ppm and 7-7.5 ppm showing the folded feature of thymosin-β4. These peaks fell into congested $^{1}$H window (7.8-8.4 ppm) (red), which exactly overlap with the peaks (blue) of the unfolded free form thymosin-β4, showing that LIM5-T displaces thymosin-β4 from actin due to the competitive binding events. (**B**) 0.1 mM $^{1}$H-$^{15}$N HSQC of α-Parvin 69-372 (α-Parvin NCH1-CH2) in the absence and presence of 0.2 mM AP-actin showing that α-Parvin NCH1-CH2 has no binding to G-actin. Comparison of Parvin N-terminal domain of WT (**C**) and mutant 5A (**D**) binding to F-actin by the high-speed F-actin co-sedimentation experiments, indicating that parvin 5A exhibiting significantly reduced binding to F-actin (**D**) vs. WT Parvin (**C**). The F-actin bound protein bands were estimated by scanning densitometry of the Coomassie Blue-stained SDS-gels. The band intensities

were normalized by background subtraction in the control lane (actin only). The normalized binding data were expressed as relative binding ratio by dividing the values of each pellet fraction by the maximum binding value at the saturation point. The binding data were plotted as a function of the protein concentration. The apparent dissociation constant ($K_D$) of Parvin (WT and 5A mutant) was estimated by one-site total binding fit model using the program Graphpad Prism. Experiments were carried out in triplicate and points represent mean ± S.E. and the averaged results are shown. (**E**) HSQC of 0.1 mM free $^{15}$N-labeled PINCH-1 LIM5-T (black) in the presence of 0.1 mM AP-actin (green) showing many binding-induced broadened peaks. The peaks come back upon adding 4 times excess unlabeled parvin-N (red) that also binds to AP-actin, demonstrating that LIM5-T became unbound to AP-actin due to the competition of parvin-N binding to AP-actin. (**F**) Sequence alignment of the C-terminus of PINCH isoforms and the N-terminus of Parvin isoforms showing that WH2 motif is conserved except γ-Parvin. The secondary structure prediction for Parvins was performed by the program PSIPRED and two potential helices are shown above the sequence. The loss-of-actin binding residues are highlighted by arrow heads.

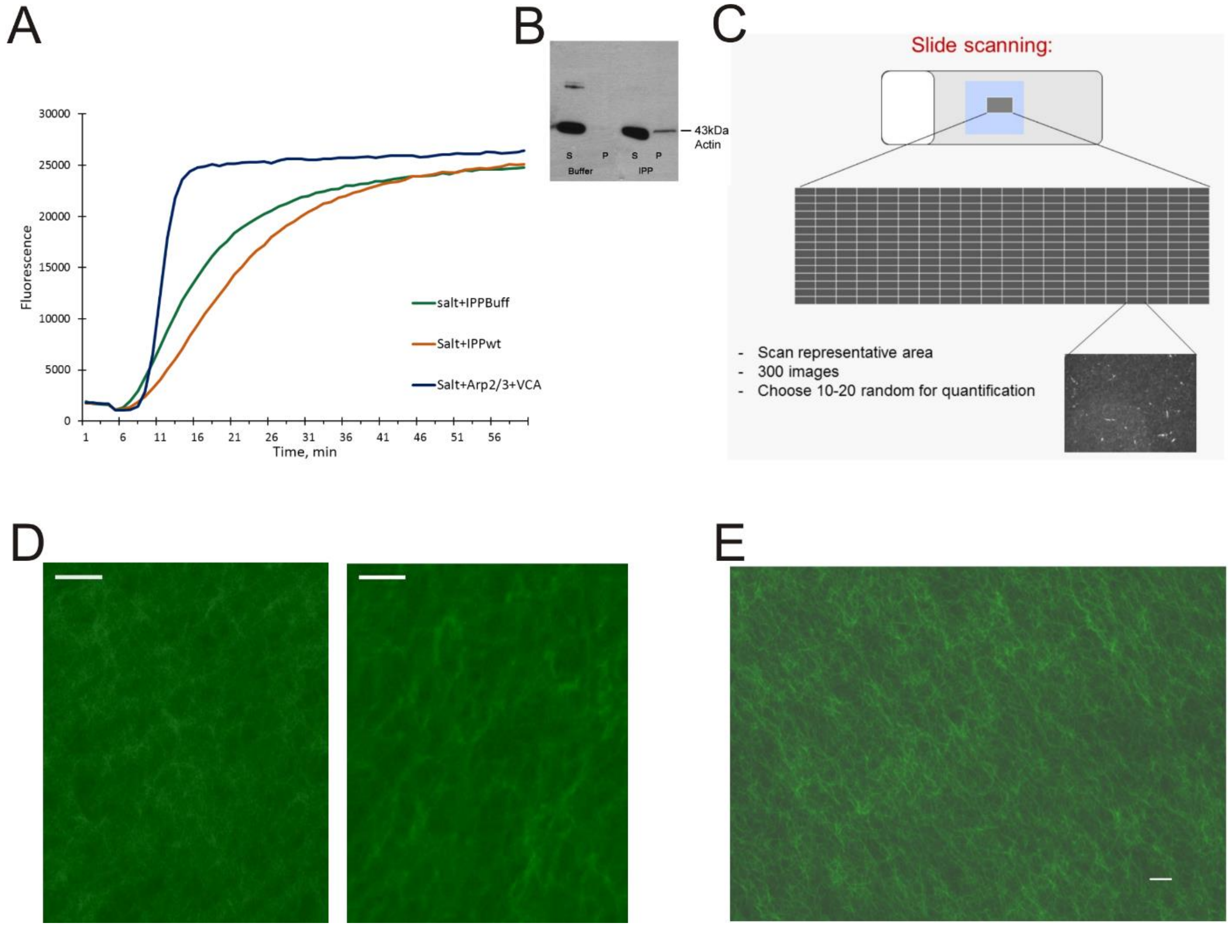


**Supplementary Figure 4. Role of IPP in actin assembly**. (**A**) Plot of the standard pyrene-based actin polymerization assay showing that IPP does not have significant effect in the actin polymerization (IPP:G-actin = 4.3 μM:4.3 μM = 1:1) as compared to Arp2/3 (Arp2/3:G-actin=30 nM:4.3 μM = 1:143). (**B**) Low speed co-sedimentation assay showing that IPP induces F-actin bundle formation. (**C**) Diagram illustrating the large-scale detection of F-actin bundles using wide field fluorescence microscopy. (**D**) Microscopic images showing that PINCH-1 (left) and α-Parvin (right) alone do not induce the F-actin bundles. Bar=100 μm. (**E**) Representative image showing that IPP-4A-5A mutations led to no F-actin bundle formation. Bar=100 μm

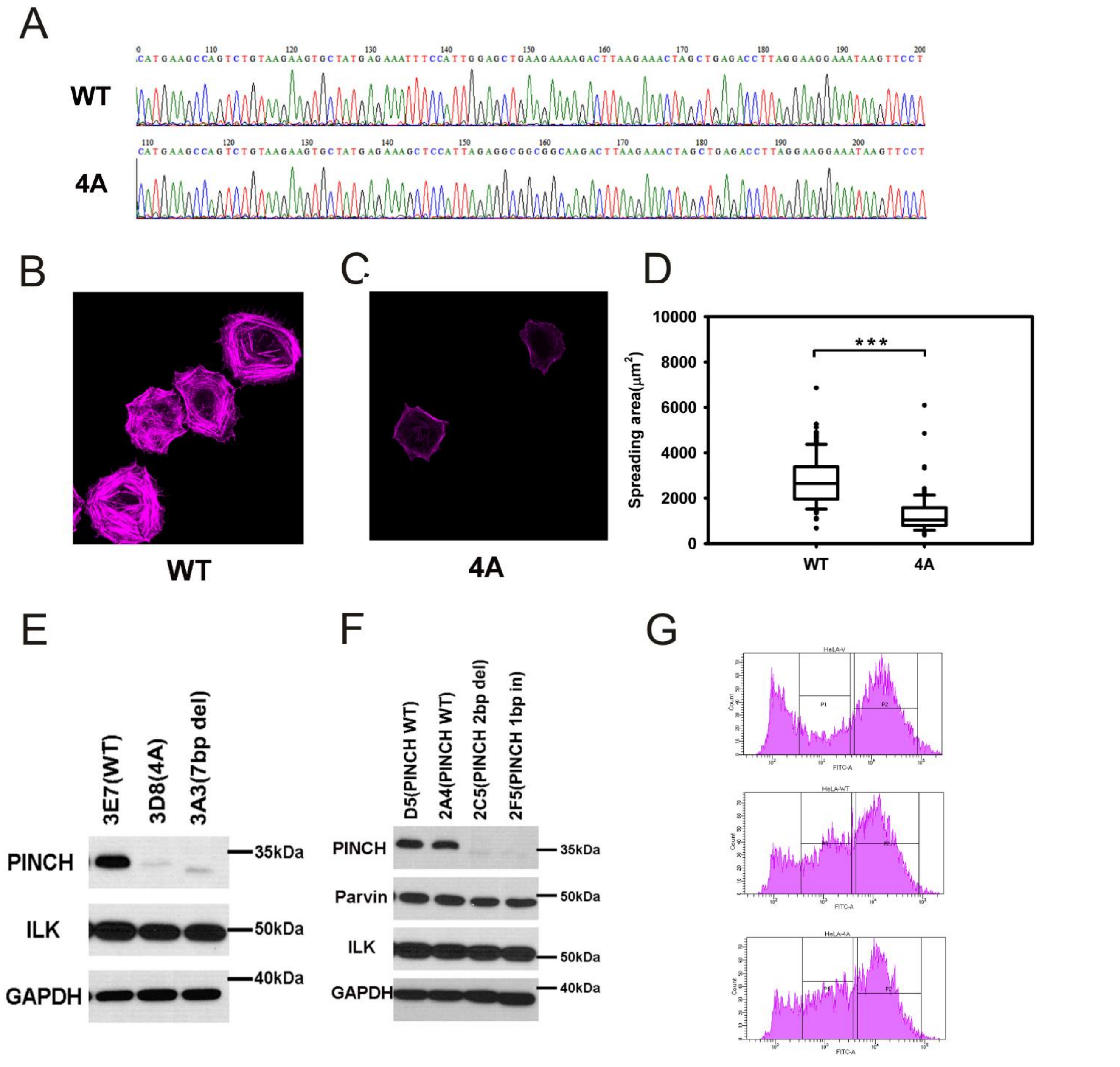


**Supplementary Figure 5. Functional analysis of PINCH1 4A mutation.** (**A**). Sequence map of PINCH WT and 4A clone generated by CRISPR. Actin staining of PINCH WT (**B**) and 4A (**C**) cells generated by CRISPR. Cells were seeded on fibronectin coated coverslips (10 µg $cm^{-2}$) for 2 h in serum reduced medium and then stained for ILK and F-actin. Images were captured by confocal microscope, under 63x magnification. (**D**) Quantification of cell spreading (118 cells each group) with Image Pro plus software. Statistics of significance were calculated by Mann-Whitney Rank Sum Test. Scale bar, 20 µm. ***, $P<0.001$. The box of the boxplot illustrates the upper and lower quartile for each population (WT and 4A). Median of spreading area is marked by a horizontal line within the box. The attached whisker indicates the range and the discrete points (•) are the outliers. (**E**) Western blot of cell lysates from HeLa clones containing PINCH1-4A. (**F**) Western blot of cell lysates from HeLa clones containing frame-shifted PINCH1-$\Delta$C. (**G**) Original sorting profile indicating that PINCH1-$\Delta$C cells with the same level of WT PINCH1 and PINCH-4A (P1 fraction) were collected for immunostaining. GFP signal level is shown on x axis. The y axis is plotted as frequency of events.

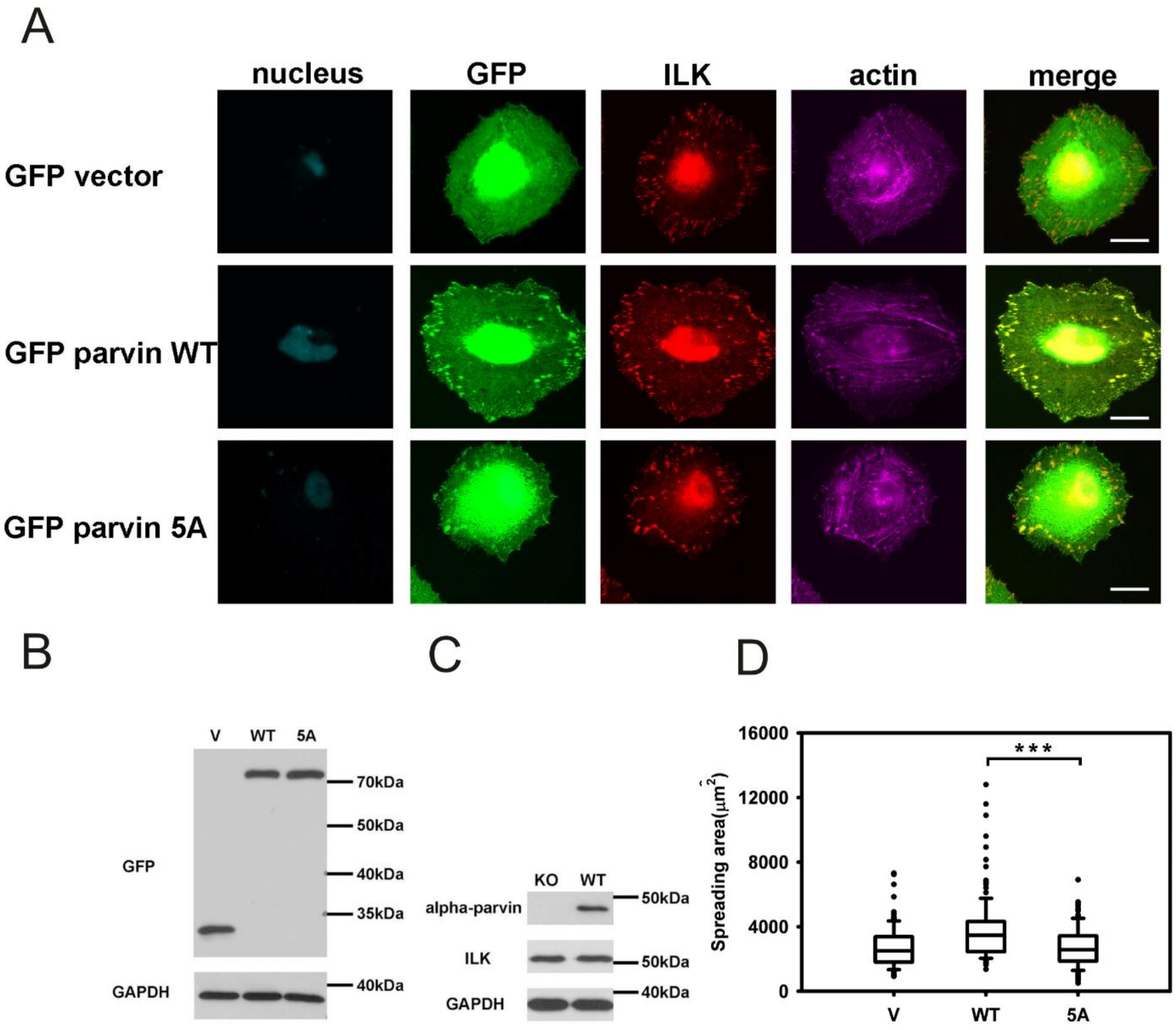


**Supplementary Figure 6. Functional analysis of α-Parvin 5A mutation**. (**A**) Recruitment of α-Parvin WT and 5A mutant to focal adhesion sites. HeLa clone with α-Parvin knockout (E1 clone) was transfected by pEGFP-c2 vector (V), pEGFP-α-Parvin wild type (WT) or pEGFP-α-Parvin 5A mutant (5A) for 24 hours and seeded on fibronectin coated coverslips (10 µg $cm^{-2}$). Cells were fixed and stained for GFP, ILK and F-actin. Images were then captured by upright wide-field fluorescence microscope, under 63x magnification. Scale bar, 20 µm. (**B**) Western blot of transfected cell lysates. (**C**) Western blot of PARVA knockout (KO) and wild type (WT) cell lysates. (**D**) Quantification of cell spreading with α-Parvin WT/5A overexpression in PARVA knockout HeLa cells (E1 clone). Spreading areas of 113 cells from each group were quantified with Image Pro Plus software based on F-actin staining. ***, $P<0.001$. Significance was generated by Kruskal-Wallis One Way Analysis of Variance on Ranks. The box of the boxplot illustrates the upper and lower quartile for each population (WT and 5A). Median of spreading area is marked by a horizontal line within the box. The attached whisker indicates the range and the discrete points (•) are the outliers.

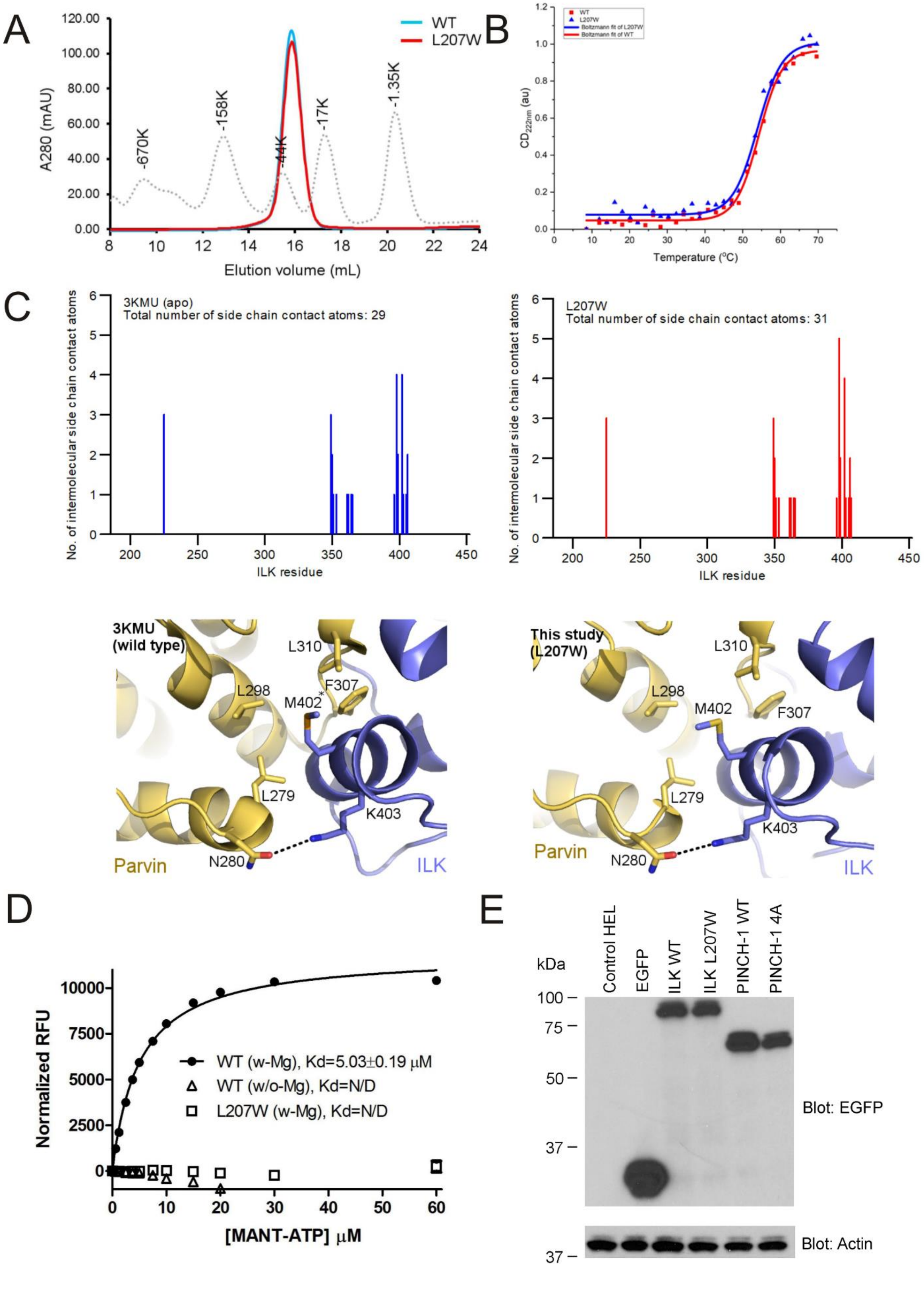
A
B
C
D
E
WT
L207W
A280 (mAU)
Elution volume (mL)
-670K
-158K
-44K
-17K
-1.35K
CD222nm (au)
Temperature (°C)
3KMU (apo)
Total number of side chain contact atoms: 29
L207W
Total number of side chain contact atoms: 31
No. of intermolecular side chain contact atoms
ILK residue
3KMU (wild type)
This study (L207W)
L310
L298
M402
F307
L279
K403
N280
Parvin
ILK
Normalized RFU
[MANT-ATP] μM
WT (w-Mg), Kd=5.03±0.19 μM
WT (w/o-Mg), Kd=N/D
L207W (w-Mg), Kd=N/D
kDa
Control HEL
EGFP
ILK WT
ILK L207W
PINCH-1 WT
PINCH-1 4A
Blot: EGFP
Blot: Actin

**Supplementary Figure 7. ILK L207W mutation effect.** (**A**). A representative elution profile of the ILK KLD wild type (WT) and the mutant (L207W) proteins on size exclusion chromatography using Superdex 200 10/300 (GE Healthcare). The elution positions of both the WT and L207W proteins are consistent. These elution profiles are overlaid to that of the standard molecular weight proteins (dot lines). (**B**) CD-based thermal shift assay showing an overlay of normalized thermal unfolding profiles of the recombinant ILK KLD/CH2 complex for the wild type (colored in red) and the L207W mutant/CH2 complex (colored in blue). Tm is 54.24 ± 0.26 $^{o}$C for WT ILK/CH2 complex and 53.90 ± 0.41 $^{o}$C for ILK L207W/CH2 complex. (**C**) Direct contact analysis between ILK and Parvin with the maximum distance cutoff set at 4.4-Å. (Top left) Summary of the contact atoms in the apo form (PDB ID 3KMU). Asterisk (*) stands for a selenomethionine-substituted methionine residue. (Top Right) Summary of the contact atoms in the L207W mutant form. (Down left) A close-up view of the intermolecular side chain contact residues between M402/K403 (ILK, apo form) and Parvin. A hydrogen bonding interaction is highlighted in dash lines. (Down right) The same close-up view in the L207W mutant structure as the apo form. (**D**) Quantification of the MANT-ATP binding to the ILK KLD (WT) and the mutant (L207W). The MANT-ATP binding to the ILK KLD (WT) depends on magnesium ion. (**E**) Western blotting showing the expression of WT ILK, ILK L207W, WT PINCH-1, and PINCH-1 4A in HEL cells.

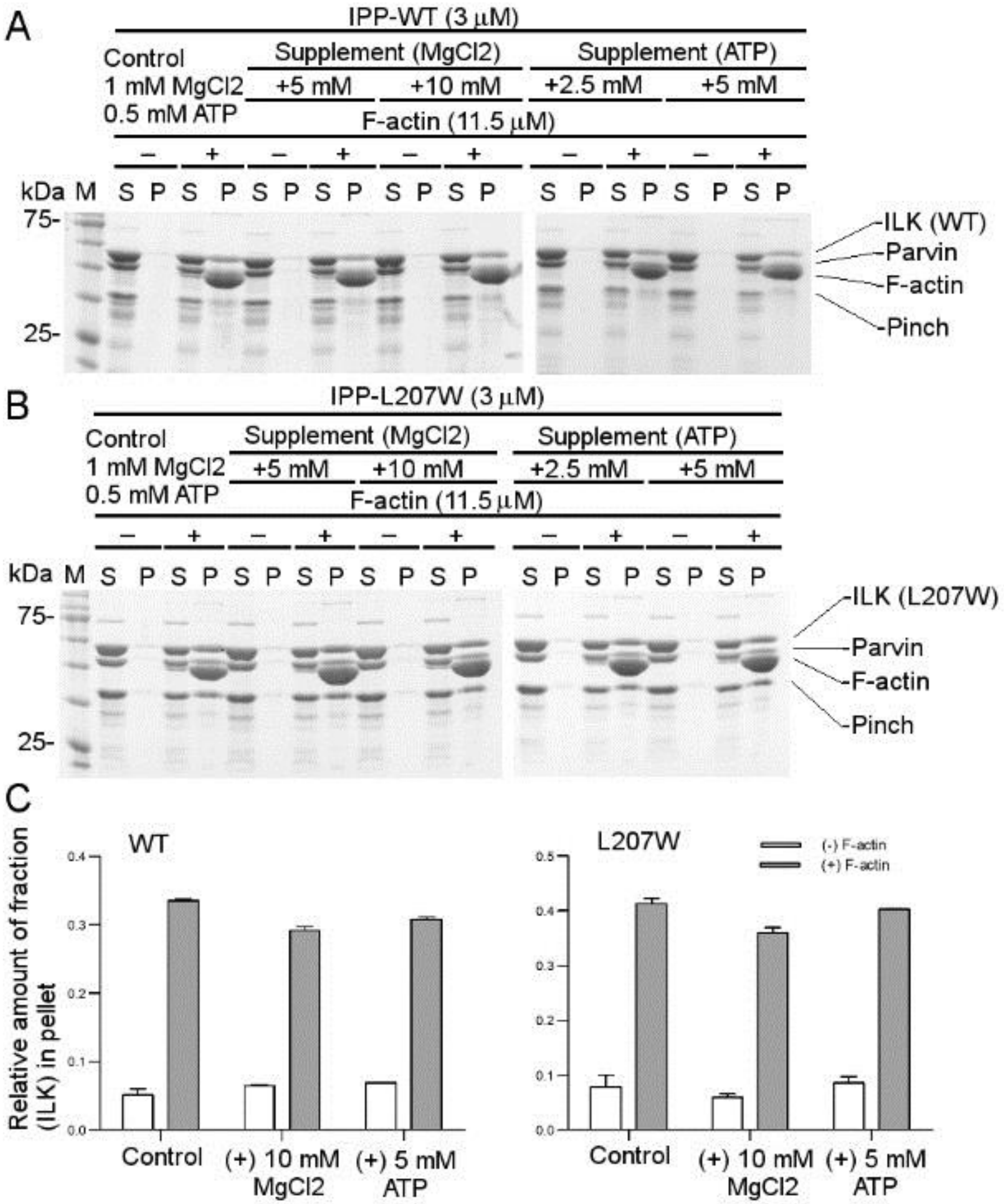


**Supplementary Figure 8. Effect of ILK L207W mutation on IPP binding to F-actin.** (**A**). SDS gel of high-speed F-actin co-sedimentation experiments of IPP at different concentrations of supplements ($MgCl_2$ or ATP). (**B**). SDS gel of high-speed F-actin co-sedimentation experiments of IPP L207W at different concentrations of $MgCl_2$ (5 mM and 10 mM) or ATP (2.5 mM and 5 mM). $MgCl_2$ and ATP in the control are 1 mM and 0.5 mM respectively. (**C**). The densitometric quantitation of ILK (wild type or L207W mutant) out of the IPP complex in pellet by the high-speed F-actin co-sedimentation experiments on effects of increasing concentrations of

supplements ($MgCl_2$ or ATP). The F-actin co-sedimentation experiments were carried out at 3 μM for both the wild type (WT) and the mutant (L207W) IPP in the 5% glycerol-containing protein buffer with supplements at two distinct concentrations of $MgCl_2$ (additional 5 mM and 10 mM) and ATP (additional 2.5 mM and 5 mM). The concentrations of $MgCl_2$ and ATP in the control were 1 mM and 0.5 mM, respectively. The supernatants and pellets of IPP in the presence/absence of F-actin were analyzed by SDS-gels, and the relative amounts of ILK (wild type or L207W mutant) at higher concentration of supplements were estimated by scanning densitometry of the Coomassie blue-stained SDS-gels. The relative amounts of fraction of ILK (as a representative protein of IPP complex) in pellets (in the absence and presence of F-actin) were calculated by dividing the pellet fraction by the total fractions (supernatant and pellet) in each condition, and shown in white (background without F-actin) and gray (with F-actin) columns, respectively. Two independent experiments were carried out, and the averaged results are shown.

**Figure 1b**

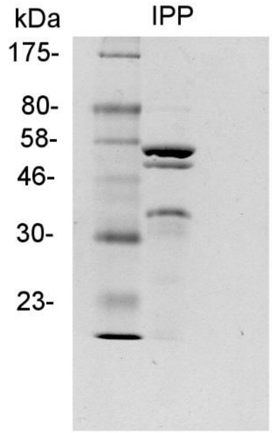


**Figure 1c**

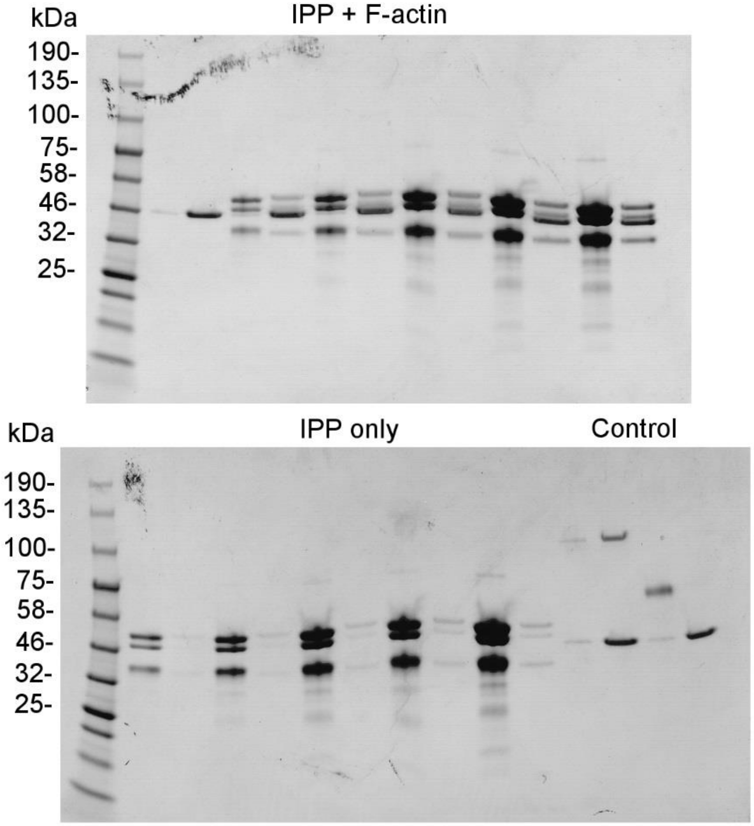


**Supplementary Figure 9**. Uncropped gel pictures for Fig. 1b and 1c.

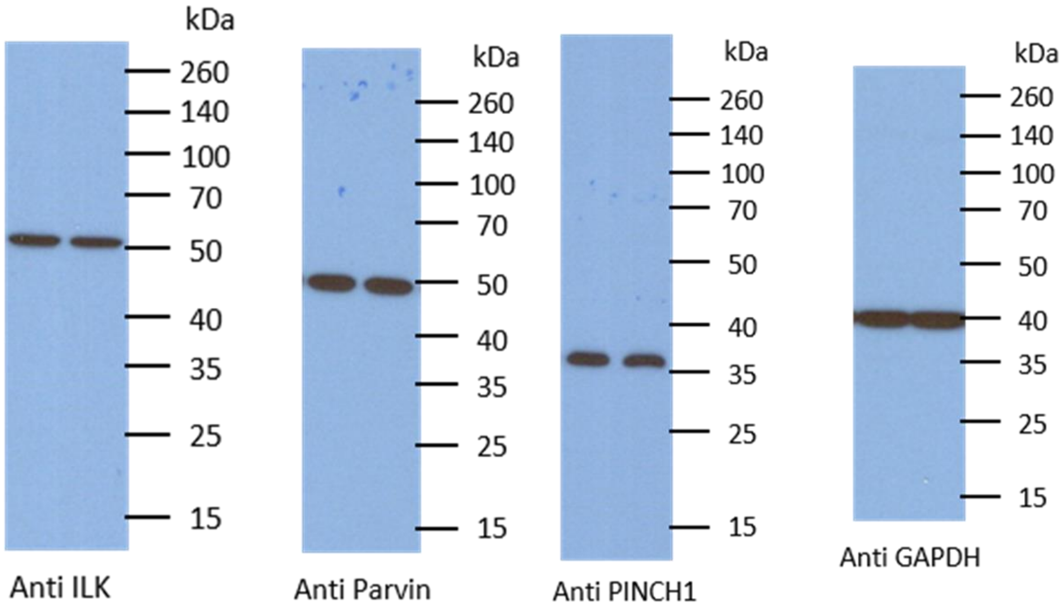


**Supplementary Figure 10** (uncropped gel pictures for Fig 8b).

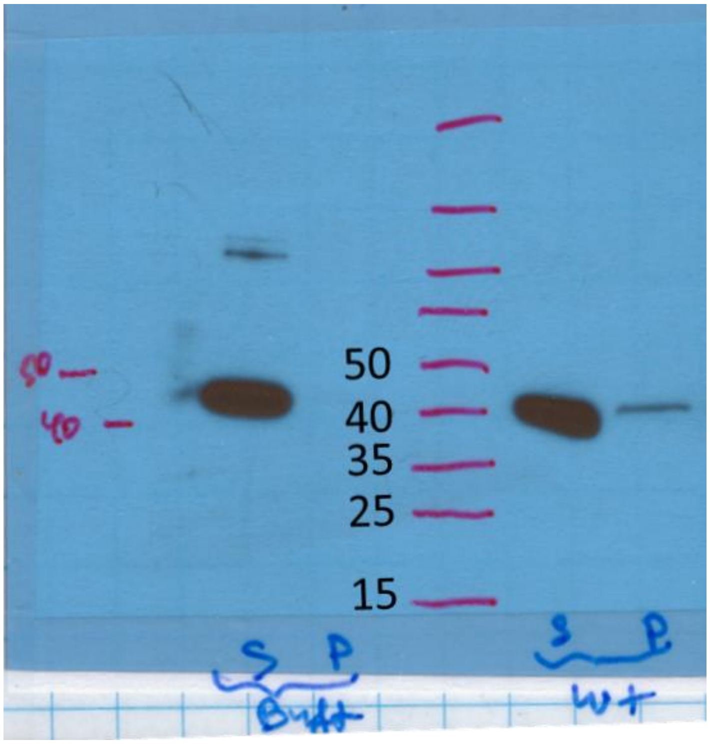


Supplementary Figure 11 (uncropped gel picture for Supplementary Figure 4b)

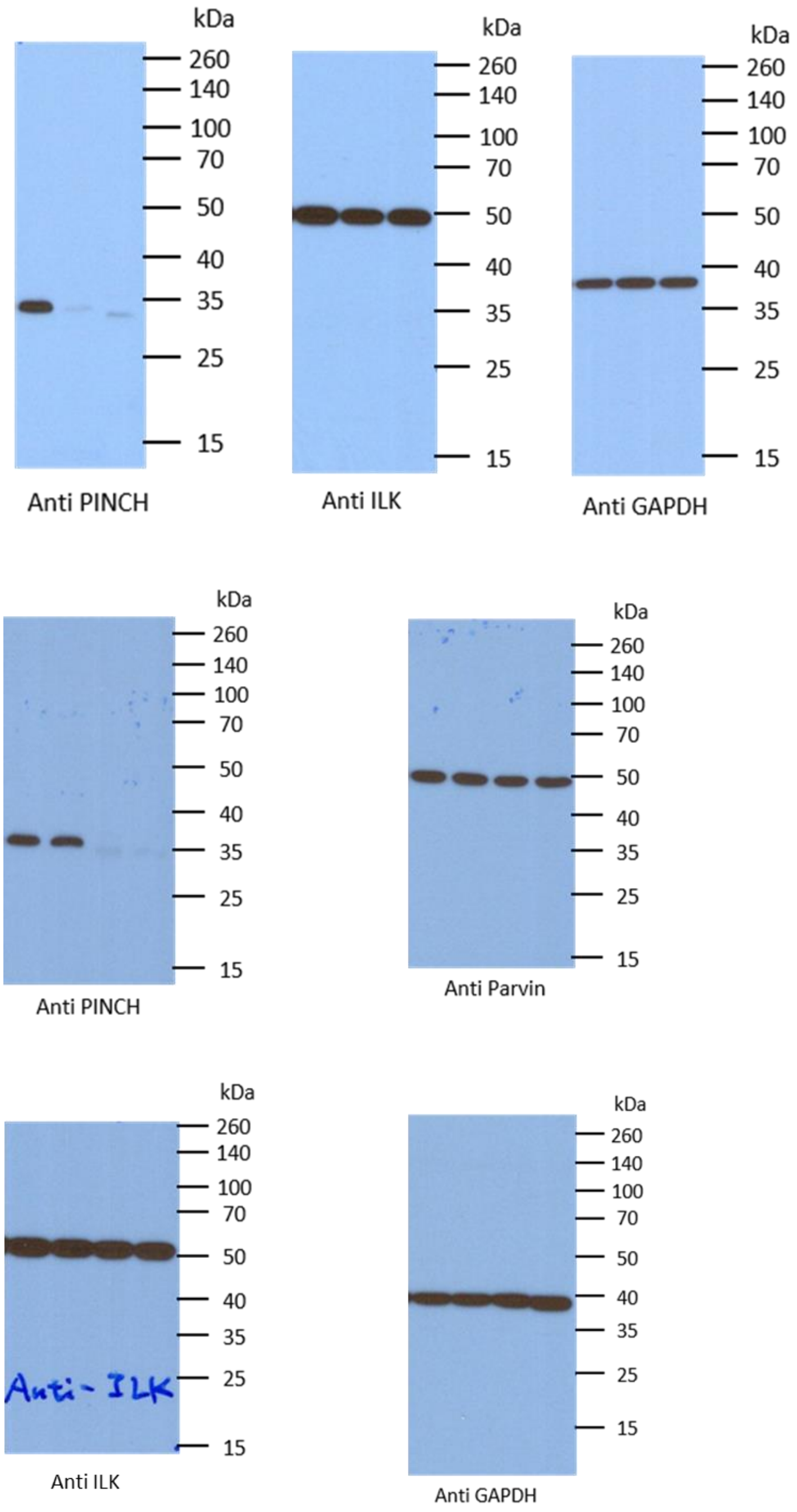


**Supplementary Figure 12** (uncropped gel pictures for Supplementary Figure 5E and 5F).

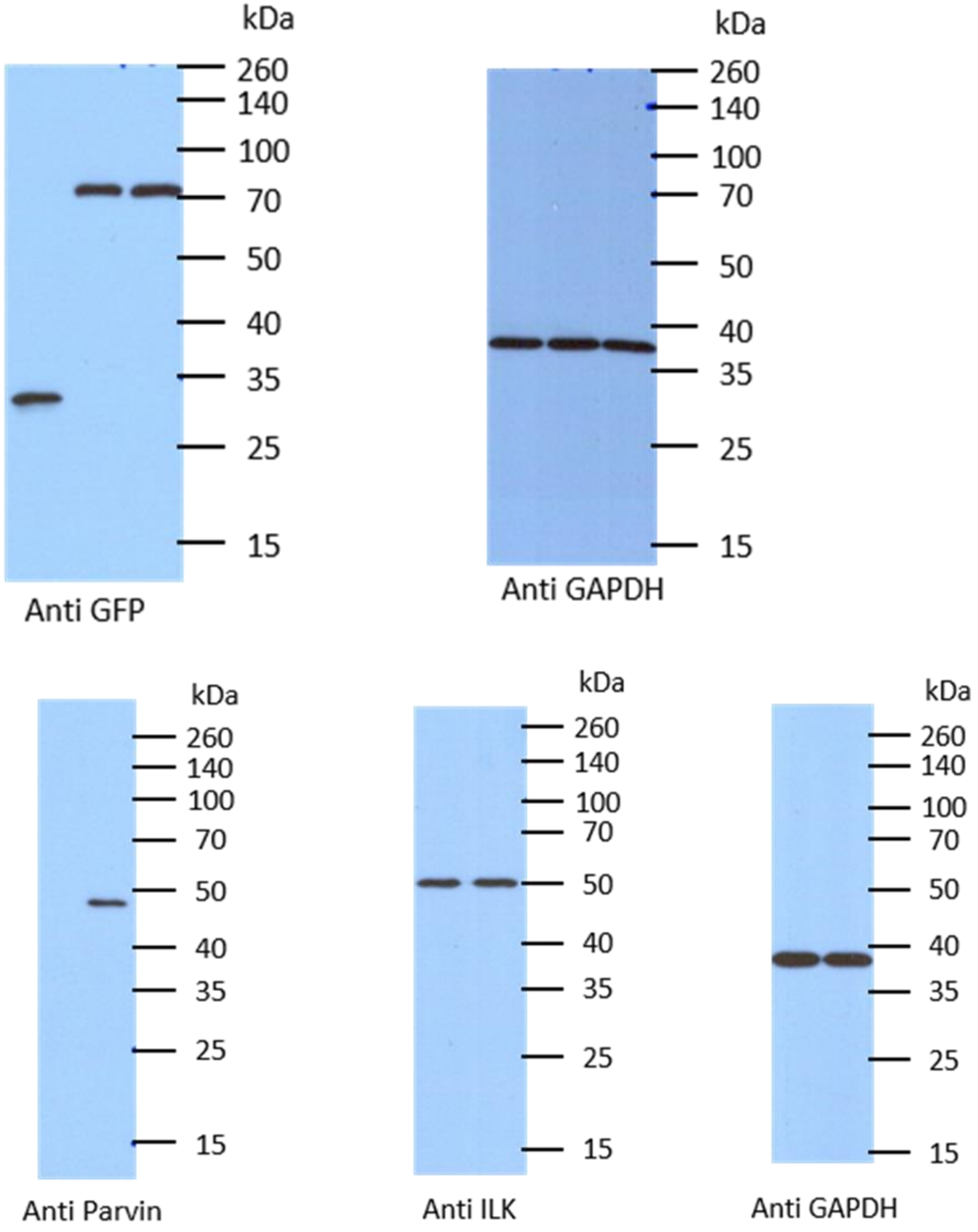


**Supplementary Figure 13** (uncropped gel pictures for Supplementary Figure 6).

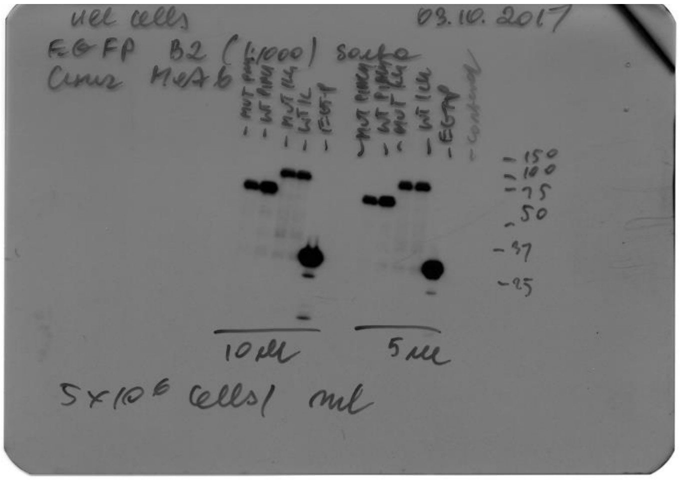

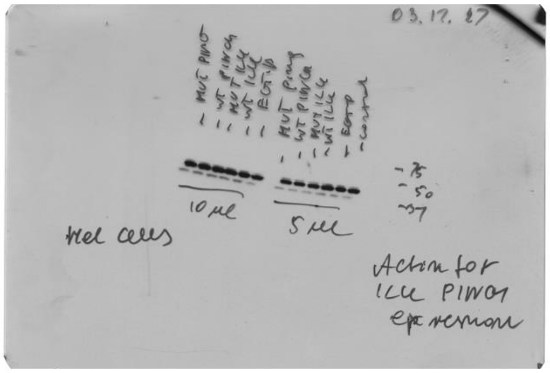

**Supplementary Figure 14** (uncropped gel pictures for Supplementary Figure 7e).

**Supplementary Table 1.**

| Reagent or resource | Source | Identifier |
|---|---|---|
| Antibodies | | |
| Chicken anti-GFP (1:2000 for ICC) | Abcam | ab13970 |
| Mouse anti-vinculin (1:400 for ICC) | Sigma | V9131 |
| Rabbit anti-ILK (1:500 for ICC, 1:5000 for WB) | Abcam | ab76468 |
| Goat anti-chicken IgY Alexa Fluor 488 (1:1000 for ICC) | Abcam | ab150173 |
| Goat anti-rabbit IgG Alexa Fluor 568 (1:1000 for ICC) | Abcam | ab175696 |
| Goat anti-mouse IgG Alexa Fluor 488 (1:1000 for ICC) | Abcam | ab150117 |
| Alexa Fluor 647 phalloidin (1:40 for ICC) | Fisher | A22287 |
| Alpha-parvin rabbit mAb (1:1000 for WB) | Cell Signaling | 8190 |
| Mouse anti-PINCH (1:1000 for WB) | BD | 612710 |
| GFP XP rabbit mAb (1:1000 for WB) | Cell Signaling | 2956 |
| GAPDH XP rabbit mAb (1:1000 for WB) | Cell Signaling | 5174 |
| Anti-rabbit IgG, HRP linked (1:3000 for WB) | Cell Signaling | 7074 |
| Anti-mouse IgG, HRP linked (1:3000 for WB) | Cell Signaling | 7076 |
| Mouse mAb anti-Actin (1:2000 for WB) | Calbiochem | CP01 |
| Bacterial, Insect, and Virus Strains | | |
| *E. coli* DH5alpha cells | Invitrogen | 18265-017 |
| *E. coli* BL21 (DE3) pLysS cells | Invitrogen | C6060-03 |
| *E. coli* BL21 (DE3) cells | Invitrogen | C6000-03 |
| Recombinant baculovirus for AP-G-actin | 1 | |
| Sf-9 insect cells | Invitrogen | 11496-015 |
| Experimental Models: Cell Lines | | |
| HeLa | ATCC | CCL-2 |
| HeLa ILK L207W | This study | ILK-2A4 |
| HeLa PINCH 4A | This study | PINCH-3D8 |
| HeLa PINCH frameshift (C terminus deletion) | This study | PINCH-2F5 |
| HeLa PARVA KO | This study | PARVA-E1 |
| HEL cells | ATCC | TIB-180 |

(continued on the next page)

## Supplementary Table 1, continued

| Oligonucleotides | |
|---|---|
| ILK, L207W, forward | IDT |
| G AAT CAC TCT GGA GAG TGG TGG AAG GGC CGC TGG | |
| ILK, L207W, reverse | IDT |
| CCA GCG GCC CTT CCA CCA CTC TCC AGA GTG ATT C | |
| Alpha-Parvin (5A), R39A, R40A, forward | IDT |
| CTC GGA GGG ACC CTG GCC GCC GCC AAG AAA GCC AAG GAG GTG | |
| Alpha-Parvin (5A), R39A, R40A, reverse | IDT |
| CAC CTC CTT GGC TTT CTT GGC GGC GGC CAG GGT CCC CTT GAG | |
| Alpha-Parvin (5A), K41A, K42A, forward | IDT |
| GGG ACC CTG GCC GCC GCC GCG GCA GCC AAG GAG GTG TCC | |
| Alpha-Parvin (5A), K41A, K42A, reverse | IDT |
| GGA CAC CTC CTT GGC TGC CGC GGC GGC GGC CAG GGT CCC | |
| Alpha-Parvin (5A), L37A, forward | IDT |
| GGG AAA CTC GGA GGG ACC GCG GCC GCC GCC GCG GCA GCC | |
| Alpha-Parvin (5A), L37A, reverse | IDT |
| GGC TGC CGC GGC GGC GGC CGC GGT CCC TCC GAG TTT CCC | |
| ILK L207W_IVT_forward | IDT |
| TAA TAC GAC TCA CTA TAG CCC TAC CTG TCC TGC AGC TA | |
| ILK L207W_IVT_reverse | IDT |
| TTC TAG CTC TAA AAC TAG CTG CAG GAC AGG TAG GG | |
| ILK_genotyping_forward | IDT |
| TTC TTC CCT AGA GCG GGC AGA GAA GAT | |
| ILK_genotyping_reverse | IDT |
| ACT GGG AGC ACA TTT GGA TGC GAG AAA ATC | |
| PINCH4A_IVT_forward | IDT |
| TAA TAC GAC TCA CTA TAG GTG CTA TGA GAA ATT TCC AT | |
| PINCH4A_IVT_reverse | IDT |
| TTC TAG CTC TAA AAC ATG GAA ATT TCT CAT AGC AC | |
| PINCH_genotyping_forward | IDT |
| CAT CAC CTA GTT GCC TTC GTT TAG TTC AG | |
| PINCH_genotyping_reverse | IDT |
| CTG TGT CCT TCC CCA ACA GCA GTT GG | |
| PARVA_IVT_forward | IDT |
| TAA TAC GAC TCA CTA TAG TCA TCT TTC TTG CGG GAC GG | |
| PARVA_IVT_reverse | IDT |
| TTC TAG CTC TAA AAC CCG TCC CGC AAG AAA GAT GA | |
| ILK L207W_ssODN | IDT |
| A*GTTAGTGGGCAAGGAAGTGGCAGCAACATTTCAAGCCTCCTAACCCCTACCTGTCCTGC | |
| AGTGGTGGAAGGGCCGCTGGCAGGGCAATGACATTG*T | |

(continued on the next page)

## Supplementary Table 1, continued

| | | |
|---|---|---|
| PINCH 4A_ssODN | IDT | |
| T*TTAGGAATAAGTTTGTGGAGTTTGACATGAAGCCAGTCTGTAAGAAGTGCTATGAGAA | | |
| AGCTCCATTAGAGGCGGCGGCAAGACTTAAGAAACTAGCTGAGACCTTAGG*A | | |
| PINCH1, F307A, forward | IDT | |
| GAAGTGCTATGAGAAAGCCCCATTGGAGCTGA | | |
| PINCH1, F307A, reverse | IDT | |
| CTTCAGCTCCAATGGGGCTTTCTCATAGCACTT | | |
| PINCH1, L311A,K312A,K313A, forward | IDT | |
| GAAATTTCCATTGGAGGCGGCGGCAAGACTTA | | |
| PINCH1, L311A,K312A,K313A, reverse | IDT | |
| GCTAGTTTCTTAAGTCTTGCCGCCGCCGCCTCCAAT | | |
| Recombinant DNA | | |
| pST39-HT-ILK KLD (wild type)-Alpha-Parvin CH2 | 2 | |
| pST39-HT-ILK KLD (C346S, C422S)-Alpha-Parvin CH2 | 2 | |
| pST39-HT-ILK KLD (C346S, C422S, L207W)-Alpha-Parvin CH2 | This study | |
| pST39-Maltose binding protein fused PINCH-1 LIM1-2-ILK | 2 | |
| pST39-HT-Alpha-Parvin-ILK (C346S, C422S)-PINCH-1, IPP-WT | This study | |
| pST39-HT-Alpha-Parvin (5A)-ILK (C346S, C422S)-PINCH-1 (4A), IPP-4A5A | This study | |
| pST39-HT-Alpha-Parvin-ILK (C346S, C422S)-PINCH-1 (4A), IPP-4A | This study | |
| pST39-HT-Alpha-Parvin-ILK (C346S, C422S, L207W)-PINCH-1, IPP-L207W | This study | |
| pGEX4T1-Alpha-Parvin (residues 1-372, full-length) | This study | |
| pGEX4T1-Alpha-Parvin-N (residues 1-89, WT) | This study | |
| pGEX4T1-Alpha-Parvin-N (residues 1-91, 5A) | This study | |
| pGEX4T1-PINCH1 LIM5-T (residues 248-325) | This study | |
| pET15b -Tb4 (full-length) | This study | |
| pMALc2x-PINCH-1 (residues 1-325, full-length) | This study | |
| pET15b-Alpha-Parvin-NCH1-CH2 (residues 69-372) | This study | |
| pET15b-Alpha-Parvin-N (residues 1-91) | This study | |
| pET15b-Alpha-Parvin CH1-CH2 (residues 89-372) | This study | |
| pEGFP-c2 vector | Clonetech | 6083-1 |
| pEGFP-c2 ILK WT | 3 | |
| pEGFP-c2 ILK L207W | This study | |
| pEGFP-c2 PINCH1 WT | 4 | |
| pEGFP-c2 PINCH1 4A | This study | |
| pEGFP-c2 alpha parvin WT | 5 | |
| pEGFP-c2 alpha parvin 5A | This study | |
| Critical Commercial Assays | | |
| GeneArt™ Precision gRNA Synthesis Kit | Fisher | A29377 |
| GeneArt™ Genomic Cleavage Detection Kit | Fisher | A24372 |
| CleanCap™ Cas9 mRNA (modified) | Trilink | L-7206 |
| Actin Binding Kit | Cytoskeleton | BK001 |
| Actin Polymerization Biochem Kit | Cytoskeleton | BK003 |

(continued on the next page)

**Supplementary Table 1, continued**

| Deposited Data | | |
|---|---|---|
| Crystal structure of ILK KLD (C346S, C422S, L207W) bound to alpha-parvin CH2 | This study | 6MIB |
| NMR structure of PINCH1 LIM5-T | This study | 6MIF |
| Crystal structure of ILK KLD (C346S, C422S) bound to alpha-parvin CH2 | 2 | 3KMU |
| NMR structure of PINCH1 LIM1 | 6 | 1G47 |
| NMR structure of PINCH1 LIM4 | 7 | 1NYP |

References


1. Joel PB, Fagnant PM, Trybus KM. (2004). Expression of a nonpolymerizable actin mutant in Sf9 cells. Biochemistry 43, 11554-11559.
2. Fukuda K, Gupta S, Chen K, Wu C, Qin J. (2009). The pseudoactive site of ILK is essential for its binding to alpha-Parvin and localization to focal adhesions. Mol. Cell, 36(5):819-830.
3. Zhang Y, Chen K, Tu Y, Velyvis A, Yang Y, Qin J, Wu C. (2002). Assembly of the PINCH-ILK-CH-ILKBP complex precedes and is essential for localization of each component to cell-matrix adhesion sites. J. Cell Sci. 115, 4777-4786.
4. Zhang Y, Guo L, Chen K, Wu C. (2002). A critical role of the PINCH-integrin-linked kinase interaction in the regulation of cell shape change and migration. J. Biol. Chem. 277, 318-326.
5. TuY, Huang Y, Zhang Y, Hua Y, Wu C. (2001). A new focal adhesion protein that interacts with integrin-linked kinase and regulates cell adhesion and spreading. J. Cell Biol. 153, 585-598.
6.Velyvis A, Yang Y, Wu C, Qin J. (2001). Solution structure of the focal adhesion adaptor PINCH LIM1 domain and characterization of its interaction with the integrin-linked kinase ankyrin repeat domain. J. Biol. Chem. 276, 4932-4939.
7. Velyvis A, Vaynberg J, Yang Y, Vinogradova O, Zhang Y, Wu C, Qin J. (2003). Structural and functional insights into PINCH LIM4 domain-mediated integrin signaling. Nat. Struct. Biol. 10, 558-564.
